# Association of dietary inflammatory index and systemic inflammatory markers with mortality risk in depressed adults: a mediation analysis of NHANES data

**DOI:** 10.3389/fnut.2024.1472616

**Published:** 2024-12-11

**Authors:** Ming Tang, Xindong Chang, Haiyan Zheng, Fanyi Zeng, Guangdong Zhang, Mingfei He, Qingqing Fang, Shiwu Yin

**Affiliations:** ^1^Department of Interventional Vascular Medicine, Hefei Hospital Affiliated to Anhui Medical University, The Second People’s Hospital of Hefei, Hefei, Anhui, China; ^2^The Fifth Clinical College of Medicine, Anhui Medical University, Hefei, Anhui, China; ^3^Department of Neurology (Sleep Disorders), The Affiliated Chaohu Hospital of Anhui Medical University, Hefei, Anhui, China

**Keywords:** dietary inflammatory index, systemic immune-inflammation index, systemic inflammation response index, depression, mortality, mediation analysis

## Abstract

**Background:**

Previous research has linked systemic inflammatory markers and the Dietary Inflammatory Index (DII) with depression. However, the relationship between DII and these markers, and their impact on mortality risk among depressed adults, remains underexplored. This study aims to explore the association between DII and systemic inflammatory markers and their mediating effect on mortality risk in adults with depression.

**Methods:**

This study analyzed data from 4,981 adults with depression in the National Health and Nutrition Examination Survey (NHANES). This study quantified dietary inflammatory potential with the DII and systemic inflammation with the Systemic Immune-Inflammation Index (SII) and Systemic Inflammation Response Index (SIRI). Cox proportional hazards regression and inverse probability weighting evaluated the impact of DII, SII, and SIRI on mortality risk in depressed adults, as well as their mediating effects. Multiple linear regression analyzed the associations between DII and SII/SIRI. Restricted cubic spline analysis explored the non-linear relationship between DII and mortality risk.

**Results:**

In adjusted regression models, DII, SII, and SIRI were significantly associated with all-cause mortality risk in depressed adults, with hazard ratios (HRs) (95% CIs) from 1.333 to 1.497 (1.051–1.233, 1.689–1.832). DII was linearly related to SII, with βs (95% CIs) from 0.001 to 0.121 (0.001–0.017, 0.001–0.224). SII significantly mediated the DII-mortality risk link, especially in males (8.07%). The DII-mortality relationship was linear (*P*_non-linear_ = 0.174), with a beneficial threshold at 1.62.

**Conclusion:**

DII and SII are associated with increased all-cause mortality risk in depressed adults. The DII-related mortality risk in depression can be partially mediated by SII, with a more pronounced effect in males.

## 1 Introduction

Depression constitutes a primary contributor to the global burden of disease stemming from psychiatric disorders due to its high prevalence, substantial disease burden, elevated suicide rates, and increased mortality, making it a significant global public health concern (1–3). Furthermore, depression is associated with various serious illnesses, including cardiovascular diseases, stroke, Alzheimer’s disease, cancer, and diabetes, collectively contributing to increased mortality rates among patients with depression (4–7). Therefore, identifying and addressing modifiable risk factors is crucial to extending the life expectancy of patients with depression.

During the COVID-19 pandemic in 2020, increased SARS-CoV-2 infection rates and reduced human mobility led to a significant rise in depression prevalence. Concurrently, elevated levels of the Systemic Immune-Inflammation Index (SII) were noted in COVID-19 patients (8, 9), suggesting inflammation as a potential shared pathophysiological mechanism between COVID-19 and depression. Previous studies have emphasized the critical roles of chronic inflammation and cell-mediated immune activation in depression pathogenesis (10, 11). Furthermore, large-scale cohort studies based on UK Biobank data have revealed that chronic inflammatory states play a key role in the complex associations between depression, various diseases, and mortality rates (12). Previous studies have confirmed that systemic inflammatory markers, such as SII and Systemic Inflammation Response Index (SIRI), are associated with increased risk of multiple diseases, including depression (13–15). However, research on the association between systemic inflammatory markers and mortality risk in patients with depression is limited. In addition, dietary patterns and components, as modifiable lifestyle factors, have been demonstrated to regulate systemic inflammation levels and influence the onset and progression of diseases. For instance, the Mediterranean diet (rich in fruits, vegetables, olive oil, whole grains, etc.) is associated with lower inflammation levels and prevention of cardiovascular disease (CVD) and metabolic syndrome (16–18). In contrast, the Western diet (high in red meat, high-fat dairy products, refined grains, etc.) is linked to higher inflammation levels, hyperlipidemia, CVD, metabolic syndrome, and cancer development (19–21). Additionally, specific nutrients, including carbohydrates, polyunsaturated fatty acids, and vitamins C/D/E, are associated with lower inflammation levels (22–24). Researchers at the University of South Carolina developed the Dietary Inflammatory Index (DII) by integrating cell culture, animal studies, and epidemiological research to assess the overall inflammatory potential of diets, and found that this index correlates with levels of inflammation (25, 26).

The aforementioned studies indicate that inflammation is not only associated with depression but also linked to an increased risk of mortality in depression patients. As a modifiable lifestyle factor, the DII can improve systemic inflammation levels in depression patients. However, the interplay between DII, systemic inflammatory markers, and depression mortality risk remains underexplored. Further investigation in this area is crucial for improving patient outcomes through dietary interventions. This study aims to explore the associations between DII, systemic inflammatory markers, and depression mortality risk using data from the National Health and Nutrition Examination Survey (NHANES) and the National Death Index (NDI) from 2005 to 2018.

## 2 Methods

### 2.1 Data sources and study population

**Figure 1:**
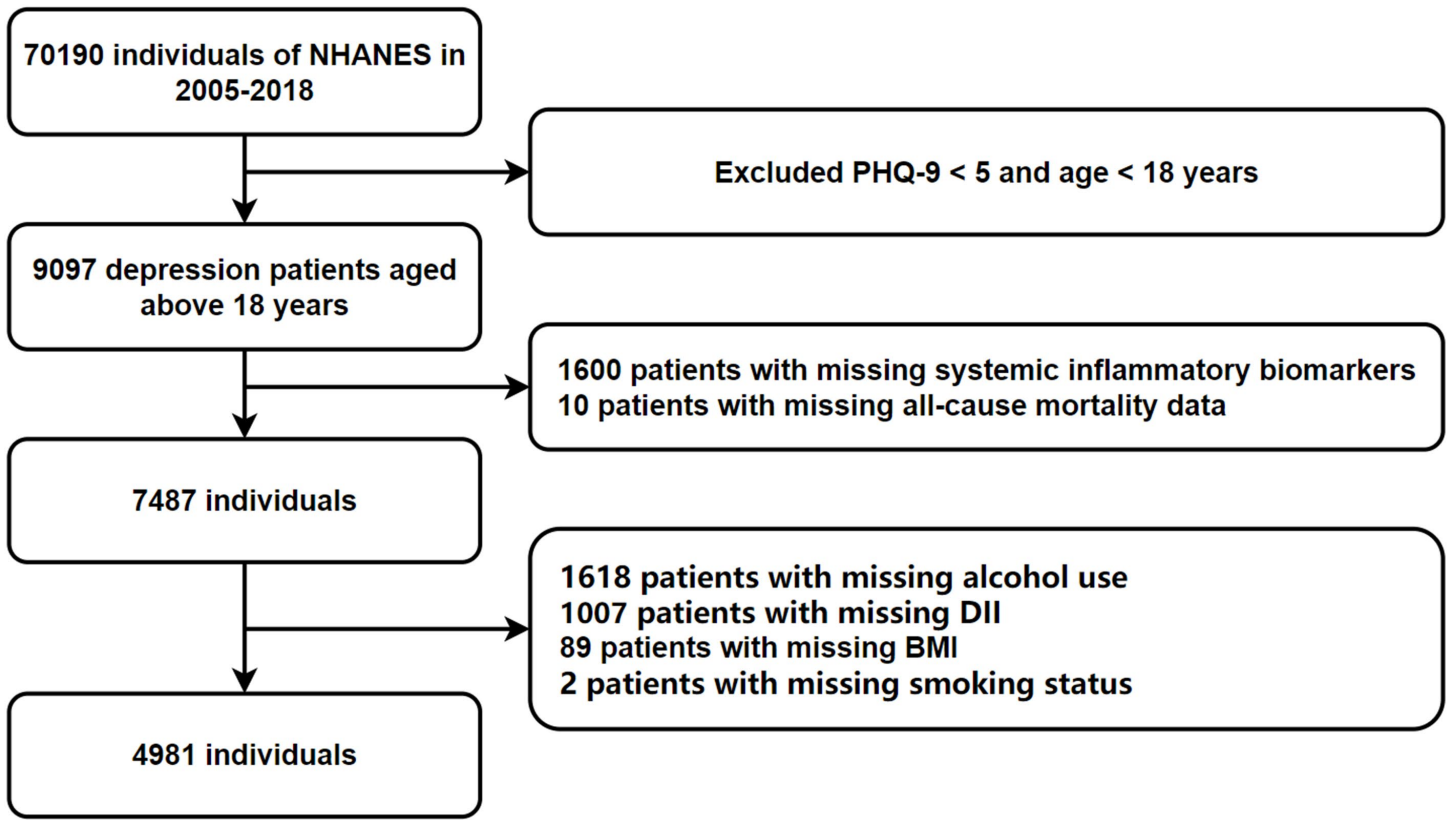
Flowchart of participant selection.

NHANES, led by the National Center for Health Statistics and part of the Centers for Disease Control and Prevention, employs a complex, multistage, probability-based cross-sectional design (27). This study utilized data from seven NHANES cycles (2005–2018), including demographic, physical, laboratory, and dietary information. Adults aged 18 and older who completed the survey were included. Participants lacking data on depression, mortality, inflammation, and the DII were excluded, resulting in a final sample of 4,981 subjects (Figure 1). Anonymized NHANES data are publicly available at www.cdc.gov/nchs/nhanes/. This study followed the guidelines in the Strengthening the Reporting of Observational Studies in Epidemiology (STROBE) Statement (Supplementary material).

### 2.2 Systemic inflammatory markers

Systemic inflammatory markers include the SII and the SIRI. SII is defined by the formula: SII = (NEUT × PLA)/LYM, where PLA represents platelet count, NEUT represents neutrophil count, and LYM represents lymphocyte count in peripheral blood. SIRI is defined by the formula: SIRI = (NEUT × MONO)/LYM, where MONO represents monocyte count (28).

### 2.3 Dietary inflammatory index

Shivappa et al. (25) developed and validated the DII by summarizing the inflammatory effect scores of 45 nutrients and estimating global means and standard deviations from 11 populations. NHANES dietary data were collected through two 24-h dietary recalls. The DII for this study was calculated using 28 nutrients, including carbohydrates, proteins, fats, vitamins, and minerals. The calculation follows five steps: (I) Assess food intake using a Food Frequency Questionnaire, 24-h dietary recall, and the Food Patterns Equivalents Database. (II) Assign an inflammation effect score to each evaluable food parameter based on existing literature. (III) Utilize dietary intake databases from various countries and regions to obtain the mean and standard deviation for each food component, calculating standardized intake scores (*Z*-scores) as follows: (daily intake of the component – global mean daily intake)/global standard deviation of daily intake (29). (IV) Multiply the *Z*-score by the inflammatory effect score of each dietary component to obtain the DII score for each component. (V) Sum the DII scores for all food components consumed in 1 day to derive the overall dietary DII score for the individual. In this study, each participant’s DII score was calculated as the average of two 24-h dietary recalls. Higher DII scores indicate a pro-inflammatory diet, while lower DII scores signify an anti-inflammatory diet.

### 2.4 Ascertainment of depression

The Patient Health Questionnaire (PHQ-9) is a nine-item tool assessing depressive symptoms over 2 weeks, based on the Diagnostic and Statistical Manual of Mental Disorders (DSM-IV) criteria (29). Administered via computer-assisted interviews, each item is scored 0–3 (“not at all” to “nearly every day”), yielding a total score from 0 to 27. Scores of 0–4 indicate no depression, while ≥5 suggests depressive symptoms, categorized as mild (5–9), moderate (10–14), and severe (15–27) (29).

### 2.5 Ascertainment of mortality

Mortality data and follow-up duration (in months) were obtained from death certificates via the NDI, from the interview date to December 31, 2019. Survival time was defined as the interval between the interview date and death or the end of follow-up. Disease-specific mortality was classified per ICD-10 guidelines: cardiovascular mortality included heart disease (I00–I09, I11, I13, I20–I51) and cerebrovascular disease (I60–I69), while cancer mortality included malignant neoplasms (C00–C97). Anonymized NDI data is freely available at https://ftp.cdc.gov/pub/Health_Statistics/NCHS/datalinkage/linked_mortality/.

### 2.6 Assessment of covariates

The covariates included in the analysis were age, sex (female or male), race/ethnicity (Non-Hispanic White, Non-Hispanic Black, Mexican American and other Hispanic and other), education level (below high school, high school or above), marital status (married, non-married), the ratio of family income to poverty (PIR), smoking status (never, current, and former), alcohol use (never, current, former), antidepressant use, body mass index (BMI), and Charlson comorbidity index (CCI). Detailed information on these covariates is provided in Supplementary Table S1 and Supplementary Information S1.

### 2.7 Statistical analyses

Participants were grouped by survival status to assess baseline characteristics. When SII, SIRI, and DII data distributions were highly skewed, natural log transformations (ln) were applied for normalization. Cox proportional hazards models (adjusted for covariates in Models 1–3) calculated hazard ratios (HR) and 95% confidence intervals (CI) for SII/SIRI (continuous, quartiles, ln) with all-cause and cause-specific mortality. Multiple linear regression models examined relationships between SII/SIRI (continuous, quartiles, ln) and DII levels. Restricted cubic splines (RCS) explored non-linear dose–response relationships between DII and mortality, identifying the optimal DII threshold. Cox models calculated associations between DII (continuous, quartiles, thresholds) and mortality under varying adjustments. Mediation analysis (1,000 bootstrap) evaluated whether inflammatory markers mediated DII’s impact on mortality. Interaction effects were assessed using *p*-values from product terms between ln inflammatory markers, DII thresholds, and covariates. Sensitivity analysis was performed using inverse probability weighting. All analyses were conducted in R studio v4.2.3 with statistical significance at *p* < 0.05. For detailed methods, see Supplementary Information S2.

## 3 Results

### 3.1 Population characteristics

A total of 70,190 potential participants completed the interviews and Mobile Examination Center screening. We excluded individuals under 18 years old, those without PHQ-9 scores, and those with scores below 5 (*n* = 61,093). Additional exclusions included participants missing data on alcohol consumption (*n* = 1,618), complete blood count (*n* = 1,600), DII (*n* = 1,007), BMI (*n* = 89), all-cause mortality (*n* = 10), and smoking status (*n* = 2). Ultimately, 4,981 participants were included in the analysis, representing approximately 28,877,823 individuals in the US after weighting. The participant selection process is illustrated in Figure 1.

Table 1 presents the baseline characteristics stratified by survival status. The cohort comprised 2074 males (41%) and 2,907 females (59%). Among patients with depression who survived, the mean SII was 564.07 ± 6.42, the mean SIRI was 1.29 ± 0.02, and the mean DII was 1.29 ± 0.04. Conversely, among deceased patients, the mean SII was 683.84 ± 28.64, the mean SIRI was 1.72 ± 0.06, and the mean DII was 1.53 ± 0.09.

**Table 1:** Characteristics of NHANES 2005–2018 participants analyzed using survival status^‡^.

Characteristic	Total	Alive	Deceased	*P*
No.	4,981	4,385	596	
Age (years)	46.90 ± 0.35	45.12 ± 0.35	63.42 ± 0.85	<0.001
Gender				0.011
Male	2074 (41%)	1756 (40%)	318 (47%)	
Female	2,907 (59%)	2,629 (60%)	278 (53%)	
Race				<0.001
Mexican	726 (7%)	693 (8%)	33 (2%)	
White	2,369 (70%)	1975 (68%)	394 (82%)	
Black	1,003 (11%)	880 (11%)	123 (11%)	
Other	883 (12%)	837 (13%)	46 (5%)	
Education				<0.001
<High school	1,418 (20%)	1,206 (19%)	212 (29%)	
≥High school	3,563 (80%)	3,179 (81%)	384 (71%)	
Marital				0.020
Married	2,112 (45%)	1880 (46%)	232 (39%)	
Non-married	2,869 (55%)	2,505 (54%)	364 (61%)	
Smoking status				0.003
Never	2,202 (43%)	2000 (43%)	202 (35%)	
Former	1,227 (25%)	1,031 (25%)	196 (30%)	
Current	1,552 (32%)	1,354 (32%)	198 (35%)	
Alcohol use				<0.001
Never	750 (12%)	642 (11%)	108 (19%)	
Former	1,023 (18%)	815 (17%)	208 (30%)	
Current	3,208 (70%)	2,928 (72%)	280 (51%)	
BMI				0.958
<30 kg/m^2^	2,713 (57%)	2,377 (57%)	336 (57%)	
≥30 kg/m^2^	2,268 (43%)	2008 (43%)	260 (43%)	
PIR	2.50 ± 0.04	2.53 ± 0.04	2.16 ± 0.09	<0.001
Depression status				0.414
Mild	3,209 (67%)	2,831 (68%)	378 (64%)	
Moderate	1,099 (21%)	959 (20%)	140 (22%)	
Major	673 (12%)	595 (12%)	78 (14%)	
Antidepressant use				0.012
Yes	1,171 (28%)	1,006 (27%)	165 (33%)	
No	3,810 (72%)	3,379 (73%)	431 (67%)	
CCI				<0.001
0	2,271 (49%)	2,125 (51%)	146 (29%)	
1–3	2069 (40%)	1793 (39%)	276 (44%)	
>3	641 (11%)	467 (10%)	174 (27%)	
PLA	254.60 ± 1.36	255.40 ± 1.30	247.10 ± 5.32	0.109
NEUT	4.57 ± 0.04	4.54 ± 0.04	4.89 ± 0.11	0.005
LYM	2.21 ± 0.01	2.23 ± 0.01	2.08 ± 0.07	0.053
MONO	0.58 ± 0.01	0.58 ± 0.01	0.62 ± 0.01	0.001
SII	575.68 ± 6.71	564.07 ± 6.42	683.84 ± 28.64	<0.001
SIRI	1.33 ± 0.02	1.29 ± 0.02	1.72 ± 0.06	<0.001
DII	1.31 ± 0.03	1.29 ± 0.04	1.53 ± 0.09	0.011

BMI, body mass index; PIR, the ratio of family income to poverty; CCI, Charlson comorbidity index; SII, systemic immune-inflammation index; SIRI, systemic inflammation response index; PLA, peripheral platelet count; LYM, lymphocyte count; MONO, monocyte count; NEUT, neutrophil count; NHANES, National Health and Nutrition Examination Survey.

‡ All estimates accounted for complex survey designs, and all percentages were weighted.

### 3.2 Association between systemic inflammation indices and depression mortality risk

The skewness of SII, SIRI, and DII were 3.30, 4.12, and −0.51, respectively, prompting natural log transformation for SII and SIRI. In Model 3, ln SII and ln SIRI were significantly associated with all-cause mortality (HR = 1.333–1.497, 95% CI 1.051–1.817) and CVD mortality (HR = 2.120–2.236, 95% CI 1.467–3.409), as were SII and SIRI as continuous variables (Table 2). Compared to the lowest quartile, the highest SIRI quartile was significantly associated with all-cause and CVD mortality, while the highest SII quartile was associated only with CVD mortality (Supplementary Table S2). No significant associations were found for cancer mortality (Table 2 and Supplementary Table S2).

**Table 2:** Hazard ratio (95% CI) for mortality risk associated with systemic inflammatory markers among depressed participants in NHANES 2005–2018.

Model	HR (95% CI)	*P*	*P* trend	Per one-unit increment in ln SII/SIRI
All-cause mortality (596/4981)				
SII				
Crude	1.001 (1.000,1.001)	<0.001	0.013	1.412 (1.092,1.824)
Model 1	1.001 (1.000,1.001)	<0.001	0.042	1.334 (1.059,1.679)
Model 2	1.000 (1.000,1.001)	<0.001	0.039	1.358 (1.070,1.724)
Model 3	1.000 (1.000,1.001)	<0.001	0.057	1.333 (1.051,1.689)
SIRI				
Crude	1.289 (1.195,1.390)	<0.001	<0.001	2.128 (1.770,2.558)
Model 1	1.137 (1.051,1.229)	0.001	<0.001	1.511 (1.256,1.818)
Model 2	1.118 (1.027,1.215)	0.010	0.002	1.523 (1.254,1.848)
Model 3	1.116 (1.027,1.214)	0.010	0.004	1.497 (1.233,1.817)
CVD mortality (179/4981)				
SII				
Crude	1.001 (1.001,1.001)	<0.001	<0.001	2.507 (1.602,3.922)
Model 1	1.001 (1.000,1.001)	<0.001	0.002	2.234 (1.455,3.429)
Model 2	1.001 (1.000,1.001)	<0.001	0.001	2.274 (1.490,3.470)
Model 3	1.001 (1.000,1.001)	<0.001	0.002	2.236 (1.467,3.409)
SIRI				
Crude	1.333 (1.213,1.464)	<0.001	<0.001	3.134 (2.253,4.359)
Model 1	1.168 (1.053,1.296)	0.003	<0.001	2.127 (1.500,3.017)
Model 2	1.150 (1.036,1.276)	0.009	<0.001	2.168 (1.522,3.088)
Model 3	1.146 (1.029,1.277)	0.013	<0.001	2.120 (1.487,3.023)
Cancer mortality (135/4981)				
SII				
Crude	1.000 (0.999,1.000)	0.770	0.243	0.821 (0.567,1.189)
Model 1	1.000 (0.999,1.000)	0.774	0.238	0.832 (0.576,1.202)
Model 2	1.000 (0.999,1.001)	0.939	0.349	0.880 (0.600,1.292)
Model 3	1.000 (1.000,1.001)	0.801	0.343	0.904 (0.609,1.340)
SIRI				
Crude	1.053 (0.968,1.146)	0.231	0.765	1.042 (0.774,1.402)
Model 1	1.033 (0.940,1.136)	0.502	0.513	1.000 (0.734,1.361)
Model 2	1.055 (0.954,1.167)	0.299	0.647	1.046 (0.764,1.432)
Model 3	1.064 (0.939,1.205)	0.334	0.503	1.031 (0.739,1.438)

SII, systemic immune-inflammation index; SIRI, systemic inflammation response index; CVD, coronary heart disease; ln: natural log-transformed; NHANES, National Health and Nutrition Examination Survey; CI, Confidence Intervals; HR, Hazard ratio.

Crude: no adjusted.

Model 1: adjusted for age (continuous), gender (male or female).

Model 2: adjusted for age (continuous), gender (male or female), race (Mexican American, non-Hispanic White, non-Hispanic Black, Other Hispanic or other races), education (less than high school, high school or above), PIR (continuous), marital (married or non-married), BMI (<30 or ≥30), smoking status (never, former or current), alcohol use (never, former or current).

Model 3: adjusted for Model 2 plus CCI (0, 1–3 or >3), antidepressant use (yes or no), depression status (mild, moderate, or major).

### 3.3 Association between systemic inflammation indices and dietary inflammation index

In Model 3, each 1 SD increase in ln SII was associated with a 0.121 rise in DII, and this linear relationship was also observed with SII as a continuous variable (Table 3). No linear association was found between ln SIRI, SIRI, and DII (*p* > 0.05, Table 3). Additionally, no significant association between the highest SII and SIRI quartiles and DII was observed in Model 3, compared to the lowest quartile of depressed patients (Supplementary Table S3).

**Table 3:** β (95% CI) for the association between systemic inflammatory markers and DII among depressed participants in NHANES 2005–2018.

	β	95% CI	*P*
SII			
Crude	0.001	(0.001, 0.001)	<0.001
Model 1	0.001	(0.001, 0.001)	0.005
Model 2	0.001	(0.001, 0.001)	0.006
Model 3	0.001	(0.001, 0.001)	0.018
ln SII			
Crude	0.190	(0.077, 0.303)	0.001
Model 1	0.137	(0.030, 0.245)	0.013
Model 2	0.134	(0.004, 0.026)	0.011
Model 3	0.121	(0.017, 0.224)	0.023
SIRI			
Crude	0.030	(−0.028, 0.089)	0.306
Model 1	0.088	(0.030, 0.147)	0.003
Model 2	0.065	(0.009, 0.121)	0.024
Model 3	0.053	(−0.003, 0.109)	0.062
ln SIRI			
Crude	0.039	(−0.057, 0.135)	0.422
Model 1	0.135	(0.040, 0.230)	0.006
Model 2	0.101	(0.011, 0.190)	0.028
Model 3	0.085	(−0.004, 0.174)	0.061

SII, systemic immune-inflammation index; SIRI, systemic inflammation response index; ln, natural log-transformed; NHANES, National Health and Nutrition Examination Survey; CI, Confidence Intervals.

Crude: no adjusted.

Model 1: adjusted for age (continuous), gender (male or female).

Model 2: adjusted for age (continuous), gender (male or female), race (Mexican American, non-Hispanic White, non-Hispanic Black, Other Hispanic or other races), education (less than high school, high school or above), PIR (continuous), marital (married or non-married), BMI (<30 or ≥30), smoking status (never, former or current), alcohol use (never, former or current).

Model 3: adjusted for Model 2 plus CCI (0, 1–3 or >3), antidepressant use (yes or no), depression status (mild, moderate, or major).

### 3.4 Association between dietary inflammatory index and depression mortality risk

**Figure 2:**
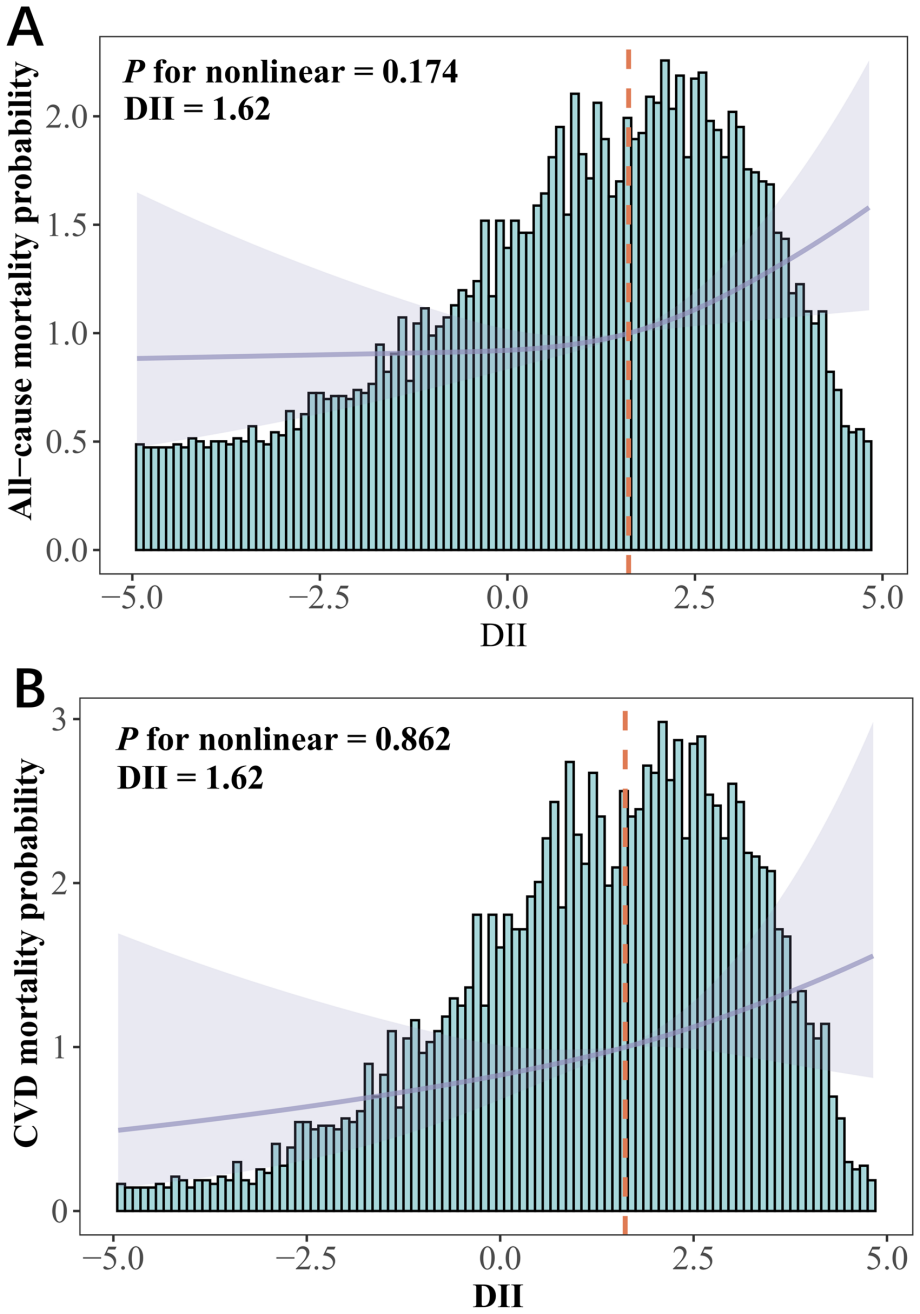
DII among depression participants in NHANES 2005–2018. **(A)** All-cause mortality and **(B)** CVD mortality risks assessed using restricted cubic spline models. HRs were adjusted for age (continuous), gender (male or female), race (Mexican American, non-Hispanic White, non-Hispanic Black, Other Hispanic or other races), education (less than high school, high school or above), PIR (continuous), marital (married or non-married), BMI (<30 or ≥30), smoking status (never, former or current), alcohol use (never, former or current), CCI (0, 1–3 or >3), antidepressant use (yes or no), depression status (mild, moderate or major), ln SII (continuous), ln SIRI (continuous).

**Figure 3:**
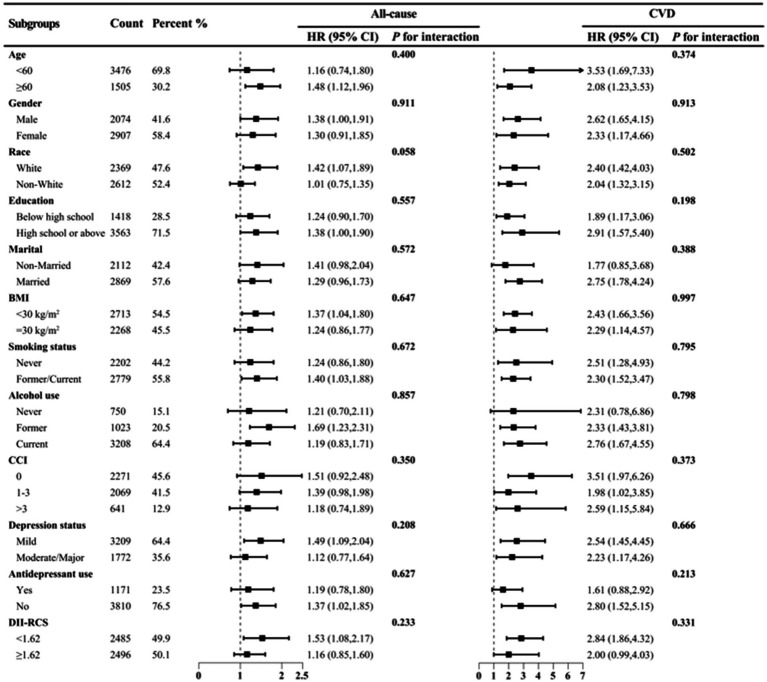
Subgroup analysis of DII (dichotomous) and ln SII with all-cause and CVD mortality risks among depression patients in NHANES 2005–2018. HRs were adjusted for age (<60 or ≥60), gender (male or female), race (White or non-White), education (less than high school, high school or above), PIR (continuous), marital (married or non-married), BMI (<30 or ≥30), smoking status (never or former/current), alcohol use (never, former or current), CCI (0, 1–3 or >3), antidepressant use (yes or no), depression status (mild or moderate/major).

In Model 3, DII was significantly associated with all-cause mortality risk (HR = 1.087, 95% CI 1.024–1.153, *P*_trend_ < 0.05) but not with CVD and cancer mortality (Table 4). Compared to the lowest quartile, the highest DII quartile showed no significant association with CVD and all-cause mortality in Model 3 but was significantly associated with cancer mortality (Supplementary Table S4). Given the significant correlation between DII and SII/ln SII, RCS regression identified the optimal DII threshold for all-cause and CVD mortality (node = 3), showing a linear relationship (*P*_non-linear_ = 0.174/0.862), with a threshold at 1.62 (estimated HR = 1) (Figure 2). When categorized, DII ≥ 1.62 was significantly associated with all-cause (HR = 1.484, 95% CI 1.202–1.832, *P*_trend_ < 0.05) and CVD mortality risk (HR = 1.548, 95% CI 1.048–2.287, *P*_trend_ < 0.05, Table 5). Subgroup analysis revealed no interaction between DII dichotomization and ln SII after covariate adjustments (Figure 3).

**Table 4:** Hazard ratio (95% CI) for mortality risk associated with DII levels among depressed participants in NHANES 2005–2018.

Model	HR (95% CI)	*P*	*P* trend
All-cause mortality (596/4981)			
Crude	1.088 (1.020,1.162)	0.010	0.010
Model 1	1.170 (1.103,1.240)	<0.001	<0.001
Model 2	1.097 (1.031,1.166)	0.004	0.004
Model 3	1.087 (1.024,1.153)	0.006	0.007
CVD mortality (179/4981)			
Crude	1.085 (0.969,1.214)	0.156	0.079
Model 1	1.159 (1.041,1.290)	0.007	0.002
Model 2	1.105 (0.982,1.243)	0.097	0.042
Model 3	1.103 (0.984,1.236)	0.094	0.041
Cancer mortality (135/4981)			
Crude	1.051 (0.930,1.187)	0.426	0.465
Model 1	1.101 (0.975,1.242)	0.120	0.137
Model 2	1.111 (0.969,1.273)	0.131	0.115
Model 3	1.122 (0.981,1.283)	0.092	0.067

DII, dietary inflammatory index; CVD, coronary heart disease; NHANES, National Health and Nutrition Examination Survey; CI, Confidence Intervals; HR, Hazard ratio.

Crude: no adjusted.

Model 1: adjusted for age (continuous), gender (male or female).

Model 2: adjusted for age (continuous), gender (male or female), race (Mexican American, non-Hispanic White, non-Hispanic Black, Other Hispanic or other races), education (less than high school, high school or above), PIR (continuous), marital (married or non-married), BMI (<30 or ≥30), smoking status (never, former or current), alcohol use (never, former or current).

Model 3: adjusted for Model 2 plus CCI (0, 1–3 or >3), antidepressant use (yes or no), depression status (mild, moderate or major), ln SII (continuous), ln SIRI (continuous).

**Table 5:** Hazard ratio (95% CI) for mortality risk associated with DII (dichotomous) among depressed participants in NHANES 2005–2018.

Model	HR (95% CI)		*P*	*P* trend
	<1.62	≥1.62		
All-cause mortality (596/4981)				
Crude	Ref	1.495 (1.215,1.840)	<0.001	0.010
Model 1	Ref	1.825 (1.486,2.241)	<0.001	<0.001
Model 2	Ref	1.513 (1.218,1.881)	<0.001	0.004
Model 3	Ref	1.484 (1.202,1.832)	<0.001	0.009
CVD mortality (179/4981)				
Crude	Ref	1.495 (1.024,2.184)	0.037	0.079
Model 1	Ref	1.798 (1.246,2.593)	0.002	0.002
Model 2	Ref	1.566 (1.048,2.339)	0.029	0.042
Model 3	Ref	1.548 (1.048,2.287)	0.028	0.042
Cancer mortality (135/4981)				
Crude	Ref	1.001 (0.681,1.470)	0.997	0.465
Model 1	Ref	1.130 (0.769,1.661)	0.534	0.137
Model 2	Ref	1.159 (0.764,1.759)	0.487	0.115
Model 3	Ref	1.254 (0.813,1.934)	0.305	0.063

DII, dietary inflammatory index; CVD, coronary heart disease; NHANES, National Health and Nutrition Examination Survey; CI, Confidence Intervals; HR, Hazard ratio.

Crude: no adjusted.

Model 1: adjusted for age (continuous), gender (male or female).

Model 2: adjusted for age (continuous), gender (male or female), race (Mexican American, non-Hispanic White, non-Hispanic Black, Other Hispanic or other races), education (less than high school, high school or above), PIR (continuous), marital (married or non-married), BMI (<30 or ≥30), smoking status (never, former or current), alcohol use (never, former or current).

Model 3: adjusted for Model 2 plu s CCI (0, 1–3 or >3), antidepressant use (yes or no), depression status (mild, moderate or major), ln SII (continuous), ln SIRI (continuous).

### 3.5 Mediation and sensitivity analyses

**Figure 4:**
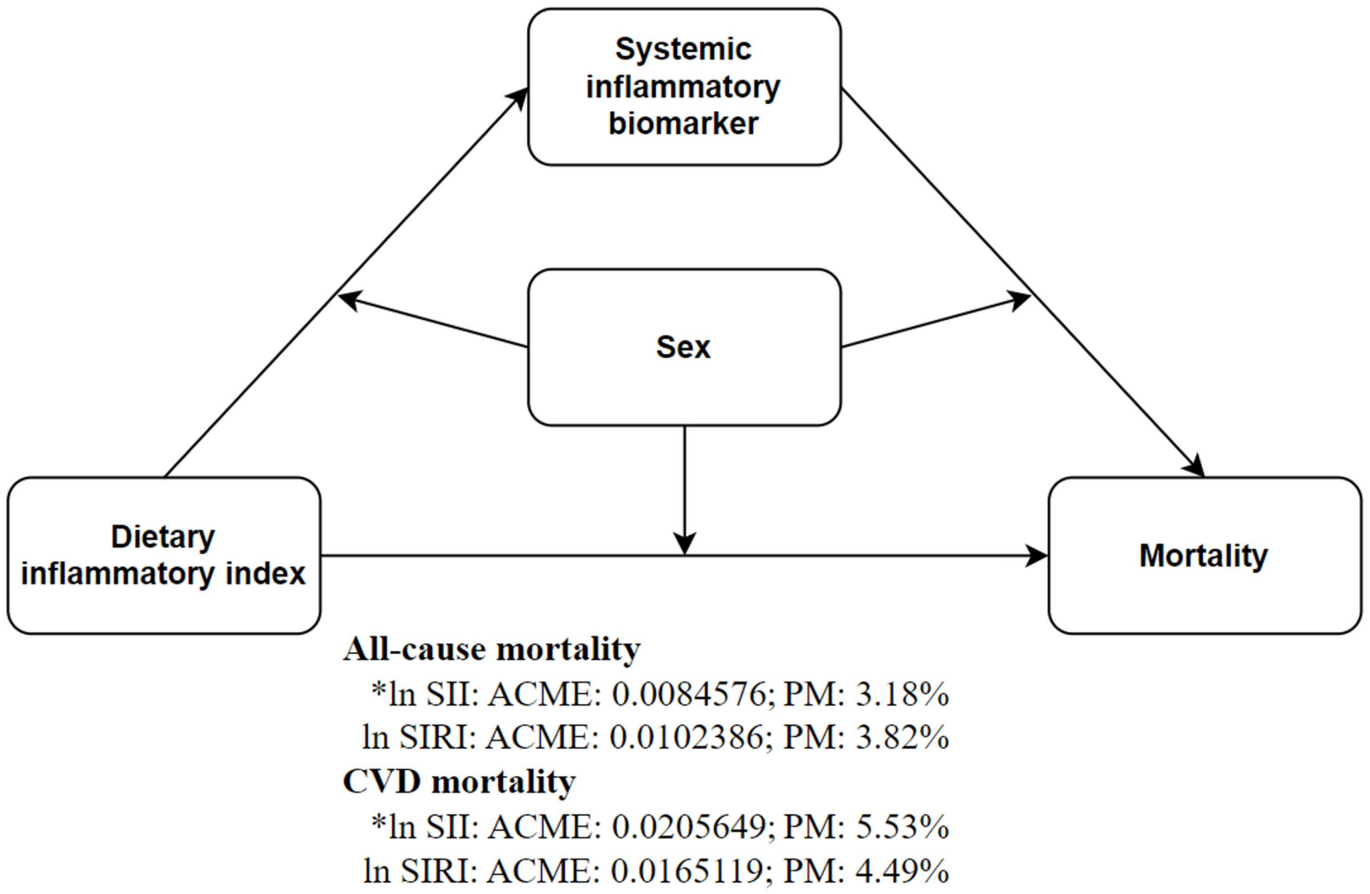
Inflammatory markers mediate the association between DII (dichotomous) and all-cause and CVD mortality risks. Mediation models were adjusted for age (continuous), gender (male or female), race (Mexican American, non-Hispanic White, non-Hispanic Black, Other Hispanic or other races), education (less than high school, high school or above), PIR (continuous), marital (married or non-married), BMI (<30 or ≥30), smoking status (never, former or current), alcohol use (never, former or current), CCI (0, 1–3 or >3), antidepressant use (yes or no), depression status (mild, moderate or major).

After adjusting for covariates, ln SII significantly mediated the relationship between DII and all-cause mortality (3.18%) and CVD mortality (5.53%) in depressed patients. Subgroup analysis by gender revealed this mediation effect was more pronounced in males, but not significant in females (Supplementary Table S5 and Figure 4). Sensitivity analysis using inverse probability-weighted Cox regression confirmed the significant association between DII dichotomization and all-cause and CVD mortality in depressed patients (*p* < 0.05, Likelihood ratio test <0.01) after covariate adjustment (Supplementary Table S6). Excluding deaths within 12 months of follow-up, DII dichotomization remained significantly associated with all-cause mortality in depressed patients (*p* < 0.05, Supplementary Table S7). Repeating the above steps after excluding CVD and cancer patients (Supplementary Tables S8–S11), the beneficial association of DII was identified with a minimum threshold at 1.57 (estimated HR = 1). When DII ≥ 1.57, it remained significantly associated with all-cause mortality risk but not with CVD risk (Supplementary Table S12 and Supplementary Figure S1). Mediation analysis further showed that ln SII/SIRI significantly mediated the association between DII dichotomization and all-cause mortality in depressed patients, particularly in males (Supplementary Table S13). Time-dependent ROC curves indicated that DII predicted 3-, 5-, and 10-year all-cause mortality with AUCs of 0.59, 0.56, and 0.54, respectively, and 0.63, 0.57, and 0.55 after excluding cancer and CVD (Supplementary Figure S2). Over time, survival rates were significantly higher in depressed patients with DII ≥ 1.62 compared to those with DII < 1.62 (*P*_log-rank_ < 0.001, Supplementary Figure S3).

## 4 Discussion

This study conducted a prospective cohort study on adult Americans with depression to explore the associations of DII, SII, and SIRI with all-cause and CVD mortality risks. Based on quartile categorization of SII/SIRI, the highest quartile of SII was significantly associated with CVD mortality risk, whereas the highest quartile of SIRI was significantly associated with both all-cause and CVD mortality risks. Further analysis revealed that ln SII/SIRI remained significantly associated with all-cause and CVD mortality risks across all models, even after excluding cardiovascular diseases and cancer. This finding has not received adequate attention in previous studies.

In contrast, Zhang et al. (30) analyzed NHANES data from 2007 to 2018 and found a significant positive association between the highest quartile of DII and both all-cause and CVD mortality risks among non-cancer depressive patients, whereas no significant association with all-cause mortality risk was observed among depressive patients with combined cancer. Our results indicate that, after adjusting for covariates, the highest quartile of DII is significantly associated with cancer-specific mortality risk among depressive patients. However, using RCS categorization, DII ≥ 1.62 was found to be significantly associated with all-cause and CVD mortality risks. A meta-analysis demonstrated that dietary patterns can significantly improve inflammatory biomarkers (31). Similarly, our study found that after adjusting for covariates, DII was not significantly associated with the highest quartiles of SII/SIRI, but showed significant associations with SII (continuous variable) and ln SII. Through RCS analysis, we identified a beneficial DII threshold of 1.62. When ln SII/SIRI was included as a covariate, DII ≥ 1.62 was significantly associated with all-cause and CVD mortality risks in depressed patients. Additionally, ln SII was found to mediate the relationship between DII and all-cause and CVD mortality risks, with this mediation effect exhibiting gender differences. However, after excluding patients who died within 12 months of follow-up, and those with CVD and cancer, the association with CVD mortality risk was no longer significant. This study also identified a significant linear dose–response relationship between DII and all-cause mortality risk. Previous research on DII and average annual weight change has reported a similar linear relationship (32). Zhang’s et al. (30) study found that the median DII for the most pro-inflammatory quartile was 2.1 and 2.2. In contrast, our study’s median DII was 1.62, and 1.58 after adjusting for confounding factors, reflecting a potential inflammatory dietary pattern among U.S. adults with depression. Modifying dietary patterns may help prevent the overall health decline following depression.

Systemic chronic inflammation is increasingly recognized as a crucial factor in the development and exacerbation of chronic diseases. A large-scale community cohort study based on the UK Biobank revealed that the primary pathways to depression-related mortality involve three disease clusters: cardiometabolic diseases, chronic inflammatory diseases, and tobacco-related diseases (12). The exploration of the relationship between mood disorders and the immune system began over two decades ago. Numerous clinical and epidemiological studies have since shown that chronic inflammation is prevalent among depression patients, potentially contributing to poor treatment response and prognosis (33–37). With the advancement of RNA sequencing technology, researchers have identified genetic variations and gene expression pathways related to inflammation in the pathogenesis of depression. Polymorphisms in genes such as IL-1β, IL-6, IL-10, TNF, MCP1/CCL2, CRP, and phospholipase A2 are among the most consistently observed findings in major depressive disorder (38). Currently, there are no large-scale clinical trials specifically investigating anti-inflammatory treatments for depression. Immune-mediated dysfunctions exhibit stage-specific characteristics, but studies have indicated significant anti-inflammatory properties and alleviation of depressive symptoms with vitamin D, selective serotonin reuptake inhibitors, and non-steroidal anti-inflammatory drugs (39–41). This suggests a potential cumulative effect between inflammation levels and depressive symptoms.

An elevated neutrophil-to-lymphocyte ratio (NLR) indicates an imbalance between innate and adaptive immune cells. Research has shown that chronic stress can lead to increased NLR, a finding confirmed in both animal models and human studies (42, 43). Neutrophils, in response to persistent stimuli, undergo a series of reactions culminating in the formation of neutrophil extracellular traps (NET). NETs serve as a bridge linking inflammation, innate immunity, oxidative stress, and cardiovascular diseases (44). Additionally, through platelet–neutrophil interactions, NET formation significantly contributes to the progression of atherosclerosis and plays a crucial role in acute coronary syndrome among patients with depression (45). These interactions localize platelets to inflammation sites, enhance reactive oxygen species production, promote thrombosis, and activate neutrophils, a key step in NET release (46, 47). Moreover, the differentiation of monocytes into macrophages or dendritic cells plays a crucial role in the progression of atherosclerosis (48). In patients with depression, activated monocytes release elevated levels of IL-6, exacerbating this process (49). Chronic inflammation not only raises the risk of CVD mortality but also heightens suicide risk among depressed individuals. A genome-wide association study based on the UK Biobank identified a link between IL-6 signaling and suicidal tendencies (34). Additionally, chronic inflammatory conditions such as asthma, osteoarthritis, and other inflammatory arthritides can further elevate mortality risk in depressed patients (12).

From a biological perspective, while extensive research has demonstrated the detrimental effects of inflammation, it is inherently a protective mechanism in the body, employed to fend off invasive harmful substances (50). This study observed a linear relationship between the DII and all-cause mortality risk in depressed patients, as demonstrated through RCS and sensitivity analyses. This linearity simplifies the interpretation of DII’s significance, highlighting its association with positive health outcomes and facilitating behavioral changes when necessary. Previous research has indicated that a DII < 2.74 can reduce depression risk (51). Our findings suggest that a DII < 1.57–1.62 can lower all-cause mortality risk in depressed patients, indicating a certain tolerance to pro-inflammatory diets among U.S. adults with depression. In multiple linear regression analyses, DII consistently exhibited a significant positive correlation with both SII and ln SII. Additionally, correlation analysis revealed significant associations between DII and various blood cell counts (Supplementary Table S14). These findings align with previous research (52), including 10 survey cycles of the NHANES study, which demonstrated a significant positive linear relationship between DII and SII (53). The positive correlation between DII and inflammatory markers suggests that dietary modifications could help reduce chronic inflammation in the body. Previous studies have shown that adherence to prudent, Mediterranean, or other anti-inflammatory diets can effectively lower levels of inflammatory markers in humans (54, 55). This study also found that a DII ≥1.62 not only directly increased the all-cause mortality risk in patients with depression but also indirectly influenced this risk through ln SII, with a more pronounced mediation effect in males (increased PM). Additionally, females had significantly higher weighted DII (1.64 ± 0.04) and SII (595.84 ± 9.29) compared to males (0.84 ± 0.05; 546.56 ± 8.08; *p* < 0.001). Mediation analysis by gender revealed an increased ADE in females across all groups. Women are at a higher risk of developing depression compared to men (56, 57), potentially due to their greater susceptibility to inflammation-induced emotional and behavioral changes (58). Conversely, depression has a more pronounced impact on mortality in men, possibly related to sex differences in telomere (59, 60). Diets with high DII may accelerate telomere shortening, which is associated with increased mortality risk (61, 62). Women generally have longer telomeres than men; although estrogen is pro-inflammatory, its response elements in the telomerase catalytic subunit promoter enhance telomerase production and activity (63–65). In addition, depression itself increases mortality rates not only due to the heightened risk of comorbid conditions like asthma and inflammatory arthritis but also from non-natural causes and medication-related risks (12, 66, 67). By comprehensively assessing dietary habits and systemic inflammation markers, early identification of health risks in depressed patients can inform tailored dietary interventions. Such interventions hold potential to enhance overall prognosis among adult depression patients in the United States.

This study has several limitations. Firstly, only 28 food parameters were used to construct the DII score, as data for the remaining 17 were unavailable. Nevertheless, the DII score remains valid when calculated with fewer than 30 nutritional parameters (68, 69). Secondly, although the data on depression and diet were validated and based on repeated measures, the use of self-reported 24-h dietary recalls and the PHQ-9 for DII and depression scores may introduce recall bias. However, the validated and repeated nature of these records helps mitigate this concern. Thirdly, despite employing inverse probability weighting and mediation analysis, the cross-sectional design of the data may limit causal inference. Future research should undertake further field intervention studies to provide stronger evidence regarding the relationship between DII and mortality risk in patients with depression.

## Data Availability

The original contributions presented in the study are included in the article/Supplementary material, further inquiries can be directed to the corresponding author.
