# Supplementary material for: Association of dietary inflammatory index and systemic inflammatory markers with mortality risk in depressed adults: a mediation analysis of NHANES data
Source: Front Nutr. 2024 Dec 11;11:1472616. doi: 10.3389/fnut.2024.1472616 (PMC11669309; doi:10.3389/fnut.2024.1472616)
Supplement: Supplementary file 1 [file Data_Sheet_1.docx]

***S******upplementary Material***

***Note*: Navigation pane allows you to quickly locate and browse**

**1. Supplementary information**

1. [Details of Covariates](#S1)

2. Details of Statistical analyses

**2. Supplementary tables**

1. **Table S1.** [The weights of each comorbidity for CCI](#TableS1)

2. **Table S2.** [HR (95% CI) for mortality risk with systemic inflammatory markers (quartiles) among depressed participants in NHANES 2005-2018](#TableS2)

3. **Table S3.** [β (95% CI) for systemic inflammatory markers (quartiles) and DII among depressed participants in NHANES 2005-2018](#TableS3)

4. **Table S4.** [HR (95% CI) for mortality risk with DII levels (quartiles) among depressed participants in NHANES 2005-2018](#TableS4)

5. **Table S5.** [Mediating effect of systemic inflammatory markers on the association between DII (dichotomous) and all-cause and CVD mortality risk](#TableS5)

6. **Table S6.** [Inverse probability weighted HR (95% CI) for mortality risk with DII (dichotomous) among depressed participants in NHANES 2005-2018](#TableS6)

7. **Table S7.** [HR (95% CI) for mortality risk with DII (dichotomous) among depressed participants with follow-up time ≥12 months](#TableS7)

8. **Table S8.** [HR (95% CI) for mortality risk with systemic inflammatory markers (quartiles) among non-cancer and non-CVD depressed participants](#TableS8)

9. **Table S9.** [HR (95% CI) for mortality risk with DII (quartiles) among non-cancer and non-CVD depressed participants](#TableS9)

10. **Table S10.** [β (95% CI) for systemic inflammatory markers and DII among non-cancer and non-CVD depressed participants](#TableS10)

11. **Table S11.** [HR (95% CI) for mortality risk associated with DII levels among non-cancer and non-CVD depressed participants in NHANES 2005-2018](#TableS11)

12. **Table S12.** [HR (95% CI) for mortality risk associated with DII (dichotomous) among non-cancer and non-CVD depressed participants in NHANES 2005-2018](#TableS12)

13. **Table S13.** [Mediating effect of systemic inflammatory markers on the association between DII (dichotomous) and all-cause mortality risk among non-cancer and non-CVD depressed participants](#TableS13)

14. **Table S14.** [Correlation between DII and blood cell parameters among NHANES 2005-2018 participants with depression](#TableS14)

**3. Supplementary figures**

1. **Figure S1.** [Restricted cubic spline model for the association between DII and all-cause mortality risk among non-cancer and non-CVD depressed participants in NHANES 2005-2018](#FigureS1)

2. **Figure S2.** [ROC curves for time-dependent all-cause mortality risk associated with DII among depression participants in NHANES 2005-2018](#FigureS2)

3. **Figure S3.** [Kaplan-Meier curves depicting all-cause mortality risk associated with DII (dichotomous) among non-cancer and non-CVD depressed participants in NHANES 2005-2018](#FigureS3)

**1. Supplementary information**

**S1.** Details of Covariates

Population demographics and socioeconomic information were gathered through structured family interviews using standardized questionnaires. Data collected included age, gender, race/ethnicity (categorized as Mexican American, non-Hispanic White, non-Hispanic Black, and other Hispanic or other races), education level (categorized as less than high school, high school or above), marital status (categorized as married or unmarried, including never married, cohabiting with partner, and other situations), poverty income ratio (PIR) as a measure of household income adjusted for specific family size thresholds, smoking status, alcohol consumption, antidepressant medication use, body mass index (BMI), and Charlson Comorbidity Index (CCI). Smoking status was categorized into three groups: "never" were individuals who had never smoked; "former" were those who had smoked ≥ 100 cigarettes in the past but had quit smoking; and "current" were individuals who had smoked ≥ 100 cigarettes in the past and were still smoking at the time of the interview. Alcohol consumption was classified as "never" (individuals who had consumed fewer than 12 drinks in their lifetime), "former" (those who had consumed ≥ 12 drinks in a year but had not drunk in the past year), and "current" (individuals who had consumed >12 drinks and continued drinking in the past year). Antidepressant medication use was defined by current use of any of the following medications (generic drug codes): AMITRIPTYLINE (d00146/d03463/d03462), BUPROPION (d00181), BUSPIRONE (d00182), CITALOPRAM (d04332), CLOMIPRAMINE (d00876), DESIPRAMINE (d00145), DOXEPIN (d00217), DULOXETINE (d05355), ESCITALOPRAM (d04812), FLUOXETINE (d00236/d04917), FLUVOXAMINE (d03804), IMIPRAMINE (d00259), MIRTAZAPINE (d04025), NEFAZODONE (d03808), NORTRIPTYLINE (d00144), PAROXETINE (d03157), PHENELZINE (d00883), PROTRIPTYLINE (d00875), SERTRALINE (d00880), TRAZODONE (d00395), VENLAFAXINE (d03181/d07113). Smoking status was categorized into three groups: "never" were individuals who had never smoked; "former" were those who had smoked ≥ 100 cigarettes in the past but had quit smoking; and "current" were individuals who had smoked ≥ 100 cigarettes in the past and were still smoking at the time of the interview. Alcohol consumption was classified as "never" (individuals who had consumed fewer than 12 drinks in their lifetime), "former" (those who had consumed ≥ 12 drinks in a year but had not drunk in the past year), and "current" (individuals who had consumed >12 drinks and continued drinking in the past year). Antidepressant medication use was defined by current use of any of the following medications (generic drug codes): AMITRIPTYLINE (d00146/d03463/d03462), BUPROPION (d00181), BUSPIRONE (d00182), CITALOPRAM (d04332), CLOMIPRAMINE (d00876), DESIPRAMINE (d00145), DOXEPIN (d00217), DULOXETINE (d05355), ESCITALOPRAM (d04812), FLUOXETINE (d00236/d04917), FLUVOXAMINE (d03804), IMIPRAMINE (d00259), MIRTAZAPINE (d04025), NEFAZODONE (d03808), NORTRIPTYLINE (d00144), PAROXETINE (d03157), PHENELZINE (d00883), PROTRIPTYLINE (d00875), SERTRALINE (d00880), TRAZODONE (d00395), VENLAFAXINE (d03181/d07113). The Body Mass Index (BMI) was calculated according to standardized protocols, dividing weight (in kilograms) by the square of height (in meters). In this study, BMI categorized weight status as "non-obese" (<30 kg/m²) and "obese" (≥30 kg/m²). The Charlson Comorbidity Index is a classical method used to assess the overall impact of comorbidities, including Diabetes mellitus, Diabetic retinopathy, Kidney failure, Kidney stones, Heart failure, Stroke, Chronic obstructive pulmonary disease, Asthma, Chronic bronchitis, Liver disease, Rheumatoid arthritis, and Cancer (Supplementary Table S1), all of which were based on self-reported information.

**S2. Details of Statistical analyses**

The NHANES dataset includes sample weights assigned to each participant to address the complexities of the survey design, such as oversampling, non-response, and post-stratification adjustments to match the total population from the Census Bureau. Participants were grouped by survival status to examine their baseline characteristics. For calculating SII, SIRI, and DII, the "skewness" function was used to assess data distribution. If skewness was outside the range of -1 to 1, natural log transformation (ln) was applied to achieve normal distribution. Using the "survey" package, weighted means ± standard errors were calculated for continuous variables, and weighted percentages for categorical variables.

Using complex sample Cox proportional hazards regression models, we calculated hazard ratios (HR) and 95% confidence intervals (CI) for the associations between SII/SIRI (continuous variables, quartiles and ln) and all-cause and cause-specific mortality under various covariate adjustments. Model 1 adjusted for age and sex. Model 2 included the variables from Model 1 plus race/ethnicity, education level, marital status, BMI, PIR, smoking status, and alcohol use. Model 3 further adjusted for CCI, depression status, and antidepressant use, and additionally incorporated DII, SII/ln SII, and SIRI/ln SIRI in subsequent analyses. Multiple linear regression models were employed to examine the relationship between SII/SIRI (continuous variables, quartiles, and ln) and DII levels. Restricted cubic spline (RCS) analysis was used to explore the nonlinear dose-response relationship between DII and all-cause and cause-specific mortality, with optimal RCS knots selected based on the Akaike information criterion (AIC) to determine the beneficial threshold associated with DII. Using complex sample Cox proportional hazards regression models, we calculated the association between DII (continuous variables, quartiles, and thresholds) and all-cause and cause-specific mortality under different covariate adjustments.

To investigate whether inflammatory markers mediate the relationship between DII and mortality, we conducted mediation analysis using 1000 bootstrap samples. Interactions were evaluated by assessing the P-value of the product term between ln-transformed inflammatory markers, DII thresholds, and stratified covariates, implemented via the "jstable" package. Sensitivity analysis was performed using inverse probability weighting with the "ipw" package. We plotted time-dependent receiver operating characteristic curve (ROC) curves with the "survivalROC" package and used the "svyjskm" function to illustrate survival rates over time for depression patients at different DII levels. All statistical analyses were performed using R studio software (v4.2.3, http://www.rproject.org/), with statistical significance set at *P* < 0.05.

**2. Supplementary tables**

**Table S1.** **The weights of each comorbidity for CC****I**

| Disease | Weight |
| --- | --- |
| Diabetes mellitus | 1 |
| Diabetic retinopathy | 2 |
| Kidney failure | 2 |
| Kidney stones | 2 |
| Heart failure | 1 |
| Stroke | 1 |
| Chronic obstructive pulmonary disease | 1 |
| Asthma | 1 |
| Chronic bronchitis | 1 |
| Liver disease | 2 |
| Rheumatoid arthritis | 1 |
| Bladder cancer | 2 |
| Bone cancer | 2 |
| Brain cancer | 2 |
| Breast cancer | 2 |
| Cervical cancer | 2 |
| Colon cancer | 2 |
| Esophageal cancer | 2 |
| Gallbladder carcinoma | 2 |
| Kidney cancer | 2 |
| Leukemia | 2 |
| Liver cancer | 2 |
| Lung cancer | 2 |
| Lymphomas | 2 |
| Melanoma | 2 |
| Nervous system cancer | 2 |
| Oral cancer | 2 |
| Ovarian cancer | 2 |
| Pancreatic cancer | 2 |
| Prostatic cancer | 2 |
| Rectal cancer | 2 |
| Skin cancer (non-melanoma) | 2 |
| Other skin cancer | 2 |
| Soft tissue cancer | 2 |
| Stomach cancer | 2 |
| Testicular cancer | 2 |
| Thyroid cancer | 2 |
| Tracheal carcinoma | 2 |
| Endometrial cancer | 2 |
| Other cancer | 2 |

**Table S2.** **HR (95% CI) for mortality risk with systemic inflammatory markers (quartiles) among depressed participants in NHANE****S 2005-2018.**

|  | HR (95%CI) | | | | *P* trend | Per one-unit increment in ln SII/SIRI |
| --- | --- | --- | --- | --- | --- | --- |
|  | Q1 | Q2 | Q3 | Q4 |  |  |
| **All-cause mortality** |  |  |  |  |  |  |
| **SII** | <348.55 | 348.55-492.62 | 492.63-697.31 | ≥697.32 |  |  |
| Crude | Ref | 0.743 (0.547,1.009) | 0.994 (0.711,1.389) | 1.358 (0.999,1.845) | 0.013 | 1.412 (1.092,1.824) |
| Model1 | Ref | 0.856 (0.643,1.140) | 1.001 (0.720,1.391) | 1.299 (0.969,1.743) | 0.042 | 1.334 (1.059,1.679) |
| Model2 | Ref | 0.892 (0.666,1.195) | 0.997 (0.705,1.409) | 1.332 (0.983,1.806) | 0.039 | 1.358 (1.070,1.724) |
| Model3 | Ref | 0.934 (0.692,1.260) | 1.010 (0.716,1.425) | 1.324 (0.969,1.808) | 0.057 | 1.333 (1.051,1.689) |
| **SIRI** | <0.72 | 0.72-1.08 | 1.09-1.61 | ≥1.62 |  |  |
| Crude | Ref | 1.106 (0.786,1.555) | 1.494 (1.069,2.088) | 2.648 (1.966,3.567) | <0.001 | 2.128 (1.770,2.558) |
| Model1 | Ref | 1.005 (0.699,1.444) | 1.113 (0.799,1.551) | 1.607 (1.178,2.191) | <0.001 | 1.511 (1.256,1.818) |
| Model2 | Ref | 1.050 (0.727,1.517) | 1.132 (0.811,1.582) | 1.598 (1.144,2.233) | 0.002 | 1.523 (1.254,1.848) |
| Model3 | Ref | 1.090 (0.749,1.587) | 1.162 (0.832,1.624) | 1.581 (1.134,2.204) | 0.004 | 1.497 (1.233,1.817) |
| **CVD mortality** |  |  |  |  |  |  |
| **SII** | <348.55 | 348.55-492.61 | 492.62-697.31 | ≥697.32 |  |  |
| Crude | Ref | 0.876 (0.514,1.495) | 1.483 (0.884,2.486) | 2.472 (1.384,4.416) | <0.001 | 2.507 (1.602,3.922) |
| Model1 | Ref | 1.051 (0.618,1.786) | 1.490 (0.901,2.463) | 2.323 (1.323,4.078) | 0.002 | 2.234 (1.455,3.429) |
| Model2 | Ref | 1.150 (0.689,1.920) | 1.544 (0.896,2.661) | 2.457 (1.405,4.297) | 0.001 | 2.274 (1.490,3.470) |
| Model3 | Ref | 1.195 (0.721,1.980) | 1.549 (0.902,2.661) | 2.446 (1.405,4.255) | 0.002 | 2.236 (1.467,3.409) |
| **SIRI** | <0.72 | 0.72-1.08 | 1.09-1.61 | ≥1.62 |  |  |
| Crude | Ref | 1.236 (0.632,2.418) | 2.403 (1.236,4.673) | 4.886 (2.739,8.715) | <0.001 | 3.134 (2.253,4.359) |
| Model1 | Ref | 1.123 (0.599,2.106) | 1.755 (0.952,3.235) | 2.884 (1.682,4.946) | <0.001 | 2.127 (1.500,3.017) |
| Model2 | Ref | 1.268 (0.668,2.406) | 1.895 (1.003,3.582) | 3.136 (1.777,5.533) | <0.001 | 2.168 (1.522,3.088) |
| Model3 | Ref | 1.281 (0.679,2.417) | 1.888 (1.017,3.504) | 3.053 (1.739,5.360) | <0.001 | 2.120 (1.487,3.023) |
| **Cancer mortality** |  |  |  |  |  |  |
| **SII** | <348.55 | 348.55-492.61 | 492.62-697.31 | ≥697.32 |  |  |
| Crude | Ref | 1.247 (0.731,2.127) | 0.428 (0.219,0.840) | 0.813 (0.424,1.559) | 0.243 | 0.821 (0.567,1.189) |
| Model1 | Ref | 1.246 (0.728,2.131) | 0.420 (0.217,0.812) | 0.812 (0.419,1.575) | 0.238 | 0.832 (0.576,1.202) |
| Model2 | Ref | 1.274 (0.718,2.259) | 0.446 (0.219,0.910) | 0.852 (0.437,1.662) | 0.349 | 0.880 (0.600,1.292) |
| Model3 | Ref | 1.154 (0.630,2.113) | 0.443 (0.219,0.894) | 0.841 (0.429,1.646) | 0.343 | 0.904 (0.609,1.340) |
| **SIRI** | <0.72 | 0.72-1.08 | 1.09-1.61 | ≥1.62 |  |  |
| Crude | Ref | 0.676 (0.293,1.559) | 0.875 (0.435,1.758) | 0.822 (0.441,1.530) | 0.765 | 1.042 (0.774,1.402) |
| Model1 | Ref | 0.664 (0.285,1.547) | 0.860 (0.426,1.736) | 0.730 (0.373,1.430) | 0.513 | 1.000 (0.734,1.361) |
| Model2 | Ref | 0.759 (0.317,1.818) | 0.956 (0.473,1.932) | 0.793 (0.390,1.614) | 0.647 | 1.046 (0.764,1.432) |
| Model3 | Ref | 0.742 (0.304,1.812) | 0.898 (0.431,1.873) | 0.743 (0.360,1.532) | 0.503 | 1.031 (0.739,1.438) |

**Abbreviations:** SII, systemic immune-inflammation index; SIRI, systemic inflammation response index; CVD: coronary heart disease; ln: natural log-transformed; NHANES, National Health and Nutrition Examination Survey; CI: Confidence Intervals; HR: Hazard Ratio.

**Crude:** no adjusted.

**Model 1:** adjusted for age (continuous), gender (male or female).

**Model 2:** adjusted for age(continuous), gender (male or female), race (Mexican American, non-Hispanic White, non-Hispanic Black, Other Hispanic or other races), education (less than high school, high school or above), PIR (continuous), marital (married or non-married), BMI (<30 or ≥30), smoking status (never, former or current), alcohol use (never, former or current).

**Model 3:** adjusted for Model 2 plus CCI (0, 1-3 or >3), antidepressant use (yes or no), depression status (mild, moderate or major).

**Table S3.** **β (95% CI) for systemic i****nflammatory markers (quartiles) and DII among depressed participants in NHANES 2005-2018.**

|  | β (95%CI) | | | | Per one-unit increment in ln  SII/SIRI |
| --- | --- | --- | --- | --- | --- |
|  | Q1 | Q2 | Q3 | Q4 |  |
| **SII** | <348.55 | 348.55-492.61 | 492.62-697.31 | ≥697.32 |  |
| Crude | Ref | 0.094 (-0.096,0.283) | 0.153 (-0.030,0.336) | 0.265 (0.073,0.456) | 0.190 (0.077,0.303) |
| Model1 | Ref | 0.073 (-0.118,0.264) | 0.120 (-0.052,0.293) | 0.202 (0.017,0.387) | 0.137 (0.030,0.245) |
| Model2 | Ref | 0.101 (-0.080,0.281) | 0.121 (-0.034,0.277) | 0.185 (0.012,0.357) | 0.134 (0.032,0.236) |
| Model3 | Ref | 0.116 (-0.070,0.301) | 0.121 (-0.036,0.278) | 0.173 (-0.003,0.349) | 0.121 (0.017,0.224) |
| **SIRI** | <0.72 | 0.72-1.08 | 1.09-1.61 | ≥1.62 |  |
| Crude | Ref | 0.003 (-0.168,0.174) | 0.204 (0.045,0.362) | 0.085 (-0.081,0.250) | 0.039 (-0.057,0.135) |
| Model1 | Ref | 0.042 (-0.132,0.216) | 0.270 (0.121,0.420) | 0.238 (0.069,0.407) | 0.135 (0.040,0.230) |
| Model2 | Ref | 0.049 (-0.119,0.217) | 0.233 (0.094,0.371) | 0.166 (0.004,0.327) | 0.101 (0.011,0.190) |
| Model3 | Ref | 0.041 (-0.128,0.209) | 0.224 (0.084,0.364) | 0.137 (-0.021,0.295) | 0.085 (-0.004,0.174) |

**Abbreviations:** SII, systemic immune-inflammation index; SIRI, systemic inflammation response index; ln: natural log-transformed; CVD: coronary heart disease; ln: natural log-transformed; NHANES, National Health and Nutrition Examination Survey; CI: Confidence Intervals.

**Crude:** no adjusted.

**Model 1:** adjusted for age (continuous), gender (male or female).

**Model 2:** adjusted for age(continuous), gender (male or female), race (Mexican American, non-Hispanic White, non-Hispanic Black, Other Hispanic or other races), education (less than high school, high school or above), PIR (continuous), marital (married or non-married), BMI (<30 or ≥30), smoking status (never, former or current), alcohol use (never, former or current).

**Model 3:** adjusted for Model 2 plu s CCI (0, 1-3 or >3), antidepressant use (yes or no), depression status (mild, moderate or major).

**Table S4. HR (95% CI) for mortality risk with DII levels (quartiles) among depressed participants in NHANES 2005-****2018.**

|  | HR (95%CI) | | | | *P* trend |
| --- | --- | --- | --- | --- | --- |
|  | Q1 (<0.31) | Q2 (0.31-1.61) | Q3 (1.62-2.71) | Q4 (≥2.72) |  |
| **All-cause mortality** |  |  |  |  |  |
| Crude | Ref | 0.706 (0.505,0.988) | 1.278 (0.956,1.707) | 1.283 (0.947,1.738) | 0.010 |
| Model1 | Ref | 0.816 (0.582,1.146) | 1.618 (1.203,2.178) | 1.732 (1.320,2.271) | <0.001 |
| Model2 | Ref | 0.779 (0.559,1.086) | 1.335 (0.979,1.822) | 1.366 (1.021,1.829) | 0.004 |
| Model3 | Ref | 0.763 (0.547,1.063) | 1.304 (0.958,1.776) | 1.313 (0.984,1.752) | 0.007 |
| **CVD mortality** |  |  |  |  |  |
| Crude | Ref | 0.897 (0.531,1.517) | 1.487 (0.884,2.501) | 1.355 (0.815,2.253) | 0.079 |
| Model1 | Ref | 1.061 (0.627,1.796) | 1.899 (1.144,3.154) | 1.794 (1.115,2.884) | 0.002 |
| Model2 | Ref | 1.018 (0.594,1.743) | 1.636 (0.926,2.891) | 1.513 (0.900,2.545) | 0.042 |
| Model3 | Ref | 1.012 (0.585,1.751) | 1.620 (0.914,2.871) | 1.485 (0.899,2.452) | 0.041 |
| **Cancer mortality** |  |  |  |  |  |
| Crude | Ref | 1.220 (0.683,2.180) | 0.911 (0.513,1.617) | 1.359 (0.798,2.315) | 0.465 |
| Model1 | Ref | 1.264 (0.710,2.251) | 1.033 (0.589,1.810) | 1.614 (0.963,2.704) | 0.137 |
| Model2 | Ref | 1.314 (0.713,2.420) | 1.068 (0.591,1.930) | 1.778 (1.010,3.130) | 0.115 |
| Model3 | Ref | 1.410 (0.764,2.603) | 1.243 (0.698,2.211) | 1.840 (1.042,3.250) | 0.067 |

**Abbreviations:** DII: dietary inflammatory index; CVD: coronary heart disease; NHANES, National Health and Nutrition Examination Survey; CI: Confidence Intervals; HR: Hazard Ratio.

**Crude:** no adjusted.

**Model 1:** adjusted for age (continuous), gender (male or female).

**Model 2:** adjusted for age(continuous), gender (male or female), race (Mexican American, non-Hispanic White, non-Hispanic Black, Other Hispanic or other races), education (less than high school, high school or above), PIR (continuous), marital (married or non-married), BMI (<30 or ≥30), smoking status (never, former or current), alcohol use (never, former or current).

**Model 3:** adjusted for Model 2 plu s CCI (0, 1-3 or >3), antidepressant use (yes or no), depression status (mild, moderate or major), ln SII (continuous), ln SIRI (continuous).

**Table S5. Mediating effect of systemic infla****mmatory markers on the association between DII (dichotomous) and all-cause and CVD mortality risk.**

|  | ACME | 95% CI | ADE | PM | *P* |
| --- | --- | --- | --- | --- | --- |
| **All-cause mortality** |  |  |  |  |  |
| **ln SII** |  |  |  |  |  |
| Crude | 0.01110894 | 0.002063732, 0.02215619 | 0.2702306 | 0.039486 | 0.036 |
| Model 1 | 0.00715985 | -0.000311835, 0.01630569 | 0.4264857 | 0.016511 | 0.122 |
| Model 2 | 0.00994788 | 0.001603837, 0.02042346 | 0.2751343 | 0.034895 | 0.048 |
| Model 3 | 0.00845760 | 0.000713504, 0.01854110 | 0.2578390 | 0.031760 | 0.026 |
| **Male-ln SII** |  |  |  |  |  |
| Crude | 0.03720164 | 0.01317117, 0.06892319 | 0.3462032 | 0.097030 | <0.001 |
| Model 1 | 0.01928598 | 0.00409057, 0.04043117 | 0.3512972 | 0.052042 | 0.012 |
| Model 2 | 0.01899294 | 0.00314352, 0.04055726 | 0.1872553 | 0.092088 | 0.024 |
| Model 3 | 0.01765426 | 0.00258981, 0.03854346 | 0.2010255 | 0.080731 | 0.030 |
| **Female-ln SII** |  |  |  |  |  |
| Crude | -0.00047378 | -0.00790441, 0.00561720 | 0.4407154 | -0.001076 | 0.934 |
| Model 1 | -0.00148828 | -0.01278203, 0.00870445 | 0.5220901 | -0.002859 | 0.820 |
| Model 2 | 0.00355176 | -0.00718603, 0.01549808 | 0.4076960 | 0.0086365 | 0.616 |
| Model 3 | 0.00232194 | -0.00786182, 0.01366522 | 0.3771234 | 0.0061193 | 0.712 |
| **ln SIRI** |  |  |  |  |  |
| Crude | 0.00028690 | -0.02156649, 0.02355290 | 0.2702306 | 0.0010606 | 0.974 |
| Model 1 | 0.01460038 | 0.00104165, 0.02993930 | 0.4264857 | 0.0331010 | 0.033 |
| Model 2 | 0.01349616 | 0.00065158, 0.02882183 | 0.2751343 | 0.0467593 | 0.032 |
| Model 3 | 0.01023855 | -0.00226283, 0.02461105 | 0.2578390 | 0.0381925 | 0.178 |
| **Male-ln SIRI** |  |  |  |  |  |
| Crude | 0.07430731 | 0.03476765, 0.11776130 | 0.3462032 | 0.1767074 | <0.001 |
| Model 1 | 0.03515804 | 0.01223175, 0.06426250 | 0.3512972 | 0.0909757 | <0.001 |
| Model 2 | 0.02770854 | 0.00662007, 0.05526377 | 0.1872553 | 0.1288986 | 0.014 |
| Model 3 | 0.02578139 | 0.00571454, 0.05223878 | 0.2010255 | 0.1136711 | 0.014 |
| **Female-ln SIRI** |  |  |  |  |  |
| Crude | -0.00722652 | -0.03260518, 0.01783615 | 0.4407154 | -0.0166706 | 0.638 |
| Model 1 | -0.00546340 | -0.02621277, 0.01508267 | 0.5220901 | -0.0105752 | 0.668 |
| Model 2 | -0.00149509 | -0.02278614, 0.02059205 | 0.4076960 | -0.0036807 | 0.910 |
| Model 3 | -0.00541498 | -0.02636191, 0.01433403 | 0.3771234 | -0.0145678 | 0.638 |
| **CVD mortality** |  |  |  |  |  |
| **ln SII** |  |  |  |  |  |
| Crude | 0.02563374 | 0.00618387, 0.04906754 | 0.3393967 | 0.0702236 | 0.034 |
| Model 1 | 0.01645217 | -0.00084195, 0.03693452 | 0.4716899 | 0.0337037 | 0.122 |
| Model 2 | 0.02325252 | 0.00454496, 0.04593081 | 0.3588207 | 0.0608588 | 0.046 |
| Model 3 | 0.02056490 | 0.00215802, 0.04234681 | 0.3510531 | 0.0553388 | 0.044 |
| **Male-ln SII** |  |  |  |  |  |
| Crude | 0.05938457 | 0.02001397, 0.10775470 | 0.3320136 | 0.1517242 | <0.001 |
| Model 1 | 0.03517968 | 0.00860498, 0.07277586 | 0.3337186 | 0.0953642 | 0.010 |
| Model 2 | 0.03584590 | 0.00813264, 0.07436186 | 0.1930667 | 0.1565921 | 0.014 |
| Model 3 | 0.03674532 | 0.00757098, 0.07577117 | 0.2553979 | 0.1257784 | 0.018 |
| **Female-ln SII** |  |  |  |  |  |
| Crude | -0.00287656 | -0.02693283, 0.02014027 | 0.5130285 | -0.0056386 | 0.844 |
| Model 1 | -0.00434384 | -0.03355992, 0.02272930 | 0.6182021 | -0.0070763 | 0.810 |
| Model 2 | 0.00928346 | -0.02005977, 0.04084123 | 0.5616470 | 0.0162602 | 0.610 |
| Model 3 | 0.00626295 | -0.02309073, 0.03716973 | 0.5368404 | 0.0115318 | 0.712 |
| **ln SIRI** |  |  |  |  |  |
| Crude | 0.00034804 | -0.02899490, 0.03038620 | 0.3393967 | 0.0010244 | 0.951 |
| Model 1 | 0.02214024 | 0.00168517, 0.04629588 | 0.4716899 | 0.0448337 | 0.031 |
| Model 2 | 0.02141467 | 0.00109085, 0.04441852 | 0.3588207 | 0.0563195 | 0.030 |
| Model 3 | 0.01651191 | -0.00391023, 0.03890720 | 0.3510531 | 0.0449224 | 0.178 |
| **Male-ln SIRI** |  |  |  |  |  |
| Crude | 0.10242510 | 0.04745757, 0.16416490 | 0.3320136 | 0.2357642 | <0.001 |
| Model 1 | 0.05761239 | 0.02044238, 0.10861250 | 0.3337186 | 0.1472216 | <0.001 |
| Model 2 | 0.04983343 | 0.01254502, 0.09752316 | 0.1930667 | 0.2051602 | 0.014 |
| Model 3 | 0.04927860 | 0.01161351, 0.09732174 | 0.2553979 | 0.1617407 | 0.014 |
| **Female-ln SIRI** |  |  |  |  |  |
| Crude | -0.00976181 | -0.04371497, 0.02314815 | 0.5130285 | -0.0193969 | 0.638 |
| Model 1 | -0.00723538 | -0.03421338, 0.01945683 | 0.6182021 | -0.0118425 | 0.668 |
| Model 2 | -0.00202644 | -0.03067146, 0.02820639 | 0.5616470 | -0.0036211 | 0.910 |
| Model 3 | -0.00782412 | -0.03705794, 0.02087789 | 0.5368404 | -0.0147900 | 0.638 |

**Abbreviations:** DII: dietary inflammatory index; CVD: coronary heart disease; ACME: average causal mediation effects; ADE: average direct effects; PM: proportion mediated.

**Crude:** no adjusted.

**Model 1:** adjusted for age (continuous), gender (male or female).

**Model 2:** adjusted for age(continuous), gender (male or female; subgroup analysis excluding gender), race (Mexican American, non-Hispanic White, non-Hispanic Black, Other Hispanic or other races), education (less than high school, high school or above), PIR (continuous), marital (married or non-married), BMI (<30 or ≥30), smoking status (never, former or current), alcohol use (never, former or current).

**Model 3:** adjusted for Model 2 plu s CCI (0, 1-3 or >3), antidepressant use (yes or no), depression status (mild, moderate or major), ln SII (continuous), ln SIRI (continuous).

**Table S6. Inverse probability weighted HR (95% CI) for mortality risk with DII (dichotomous) among depress****ed participants in NHANES 2005-2018**

| Model | HR (95% CI) | | *P* | Likelihood ratio test |
| --- | --- | --- | --- | --- |
|  | <1.62 | ≥1.62 |  |  |
| **All-cause mortality**  **(596/4981)** |  |  |  |  |
| Crude | Ref | 1.329 (1.117,1.582) | 0.001 | <0.001 |
| Model 1 | Ref | 1.558 (1.300,1.868) | <0.001 | <0.001 |
| Model 2 | Ref | 1.318 (1.096,1.587) | 0.003 | <0.001 |
| Model 3 | Ref | 1.323 (1.098,1.593) | 0.003 | <0.001 |
| **CVD mortality**  **(179/4981)** |  |  |  |  |
| Crude | Ref | 1.361 (0.991,1.870) | 0.057 | 0.002 |
| Model 1 | Ref | 1.561 (1.135,2.146) | 0.006 | <0.001 |
| Model 2 | Ref | 1.380 (0.997,1.909) | 0.052 | <0.001 |
| Model 3 | Ref | 1.404 (1.003,1.966) | 0.048 | <0.001 |

**Abbreviations:** DII: dietary inflammatory index; CVD: coronary heart disease; NHANES, National Health and Nutrition Examination Survey; CI: Confidence Intervals; HR: Hazard Ratio.

**Crude:** no adjusted.

**Model 1:** adjusted for age (continuous), gender (male or female).

**Model 2:** adjusted for age(continuous), gender (male or female), race (Mexican American, non-Hispanic White, non-Hispanic Black, Other Hispanic or other races), education (less than high school, high school or above), PIR (continuous), marital (married or non-married), BMI (<30 or ≥30), smoking status (never, former or current), alcohol use (never, former or current).

**Model 3:** adjusted for Model 2 plu s CCI (0, 1-3 or >3), antidepressant use (yes or no), depression status (mild, moderate or major), ln SII (continuous), ln SIRI (continuous).

**Table S7. HR (95% CI) for mortality risk with DII (dichotomous) among depressed participants with follow****-up time ≥12 months**

| Model | HR (95% CI) | | *P* | *P* trend |
| --- | --- | --- | --- | --- |
|  | <1.62 | ≥1.62 |  |  |
| **All-cause mortality**  **(547/4931)** |  |  |  |  |
| Crude | Ref | 1.374 (1.100,1.716) | 0.005 | 0.074 |
| Model 1 | Ref | 1.693 (1.358,2.110) | <0.001 | <0.001 |
| Model 2 | Ref | 1.398 (1.102,1.775) | 0.006 | 0.036 |
| Model 3 | Ref | 1.376 (1.091,1.737) | 0.007 | 0.053 |
| **CVD mortality**  **(162/4931)** |  |  |  |  |
| Crude | Ref | 1.286 (0.837,1.976) | 0.252 | 0.409 |
| Model 1 | Ref | 1.564 (1.037,2.360) | 0.033 | 0.039 |
| Model 2 | Ref | 1.349 (0.856,2.127) | 0.197 | 0.259 |
| Model 3 | Ref | 1.330 (0.853,2.074) | 0.209 | 0.287 |
| **Cancer mortality**  **(125/4931)** |  |  |  |  |
| Crude | Ref | 0.961 (0.650,1.422) | 0.843 | 0.703 |
| Model 1 | Ref | 1.076 (0.733,1.579) | 0.708 | 0.273 |
| Model 2 | Ref | 1.116 (0.734,1.695) | 0.608 | 0.211 |
| Model 3 | Ref | 1.214 (0.789,1.869) | 0.378 | 0.128 |

**Abbreviations:** DII: dietary inflammatory index; CVD: coronary heart disease; CI: Confidence Intervals; HR: Hazard Ratio.

**Crude:** no adjusted.

**Model 1:** adjusted for age (continuous), gender (male or female).

**Model 2:** adjusted for age(continuous), gender (male or female), race (Mexican American, non-Hispanic White, non-Hispanic Black, Other Hispanic or other races), education (less than high school, high school or above), PIR (continuous), marital (married or non-married), BMI (<30 or ≥30), smoking status (never, former or current), alcohol use (never, former or current).

**Model 3:** adjusted for Model 2 plu s CCI (0, 1-3 or >3), antidepressant use (yes or no), depression status (mild, moderate or major), ln SII (continuous), ln SIRI (continuous).

**Table S8. HR (95% CI) for mo****rtality risk with systemic inflammatory markers (quartiles) among non-cancer and non-CVD depressed participants**

|  | HR (95%CI) | | | | *P* trend | Per one-unit increment  in ln SII/SIRI |
| --- | --- | --- | --- | --- | --- | --- |
|  | Q1 | Q2 | Q3 | Q4 |  |  |
| **All-cause mortality** |  |  |  |  |  |  |
| **SII** | <346.81 | 346.82-485.17 | 485.18-687.70 | ≥687.71 |  |  |
| Crude | Ref | 0.668 (0.440,1.014) | 0.976 (0.644,1.479) | 1.269 (0.881,1.827) | 0.054 | 1.363 (1.006,1.846) |
| Model1 | Ref | 0.738 (0.499,1.090) | 0.981 (0.647,1.489) | 1.246 (0.883,1.758) | 0.080 | 1.331 (1.016,1.744) |
| Model2 | Ref | 0.763 (0.514,1.133) | 0.986 (0.645,1.507) | 1.250 (0.862,1.811) | 0.106 | 1.342 (1.019,1.767) |
| Model3 | Ref | 0.770 (0.518,1.145) | 1.001 (0.656,1.527) | 1.254 (0.869,1.809) | 0.095 | 1.340 (1.030,1.743) |
| **SIRI** | <0.69 | 0.70-1.05 | 1.06-1.55 | ≥1.56 |  |  |
| Crude | Ref | 1.156 (0.721,1.854) | 1.607 (1.038,2.486) | 2.359 (1.622,3.430) | <0.001 | 1.903 (1.537,2.356) |
| Model1 | Ref | 1.065 (0.655,1.732) | 1.222 (0.800,1.867) | 1.626 (1.082,2.445) | 0.009 | 1.474 (1.175,1.849) |
| Model2 | Ref | 1.131 (0.707,1.809) | 1.175 (0.769,1.796) | 1.576 (1.037,2.394) | 0.025 | 1.444 (1.145,1.822) |
| Model3 | Ref | 1.169 (0.731,1.869) | 1.204 (0.791,1.833) | 1.589 (1.054,2.397) | 0.021 | 1.444 (1.150,1.813) |
| **CVD mortality** |  |  |  |  |  |  |
| **SII** | <346.81 | 346.82-485.17 | 485.18-687.70 | ≥687.71 |  |  |
| Crude | Ref | 1.133 (0.478,2.685) | 2.019 (0.988,4.126) | 2.910 (1.328,6.377) | 0.005 | 2.352 (1.502,3.685) |
| Model1 | Ref | 1.297 (0.541,3.110) | 2.001 (0.977,4.097) | 2.780 (1.279,6.042) | 0.008 | 2.180 (1.428,3.330) |
| Model2 | Ref | 1.333 (0.588,3.024) | 2.024 (0.966,4.242) | 2.853 (1.292,6.300) | 0.011 | 2.123 (1.378,3.271) |
| Model3 | Ref | 1.335 (0.589,3.025) | 2.026 (0.964,4.259) | 2.850 (1.299,6.253) | 0.010 | 2.126 (1.397,3.236) |
| **SIRI** | <0.69 | 0.70-1.05 | 1.06-1.55 | ≥1.56 |  |  |
| Crude | Ref | 1.079 (0.473,2.459) | 2.316 (0.937,5.725) | 4.027 (1.799,9.013) | <0.001 | 2.632 (1.781,3.891) |
| Model1 | Ref | 0.972 (0.449,2.103) | 1.675 (0.716,3.916) | 2.659 (1.258,5.620) | 0.004 | 1.956 (1.316,2.907) |
| Model2 | Ref | 1.008 (0.452,2.250) | 1.614 (0.695,3.751) | 2.669 (1.256,5.670) | 0.004 | 1.883 (1.284,2.761) |
| Model3 | Ref | 1.012 (0.455,2.249) | 1.613 (0.697,3.729) | 2.665 (1.260,5.634) | 0.004 | 1.885 (1.280,2.776) |
| **Cancer mortality** |  |  |  |  |  |  |
| **SII** | <346.81 | 346.82-485.17 | 485.18-687.70 | ≥687.71 |  |  |
| Crude | Ref | 1.018 (0.514,2.014) | 0.239 (0.090,0.635) | 0.570 (0.250,1.300) | 0.068 | 0.659 (0.428,1.015) |
| Model1 | Ref | 1.020 (0.505,2.061) | 0.243 (0.091,0.648) | 0.578 (0.253,1.317) | 0.069 | 0.669 (0.431,1.038) |
| Model2 | Ref | 1.053 (0.491,2.257) | 0.250 (0.084,0.742) | 0.592 (0.258,1.360) | 0.116 | 0.704 (0.430,1.153) |
| Model3 | Ref | 1.013 (0.447,2.298) | 0.268 (0.094,0.760) | 0.655 (0.278,1.543) | 0.169 | 0.787 (0.450,1.378) |
| **SIRI** | <0.69 | 0.70-1.05 | 1.06-1.55 | ≥1.56 |  |  |
| Crude | Ref | 0.643 (0.218,1.897) | 0.959 (0.399,2.304) | 0.642 (0.286,1.442) | 0.420 | 0.926 (0.630,1.362) |
| Model1 | Ref | 0.644 (0.213,1.944) | 0.985 (0.408,2.375) | 0.588 (0.251,1.376) | 0.344 | 0.915 (0.617,1.357) |
| Model2 | Ref | 0.753 (0.263,2.162) | 1.104 (0.470,2.593) | 0.693 (0.278,1.725) | 0.539 | 0.995 (0.649,1.525) |
| Model3 | Ref | 0.767 (0.269,2.189) | 1.077 (0.448,2.590) | 0.726 (0.270,1.955) | 0.640 | 1.045 (0.642,1.702) |

**Abbreviations:** SII, systemic immune-inflammation index; SIRI, systemic inflammation response index; CVD: coronary heart disease; ln: natural log-transformed; CI: Confidence Intervals; HR: Hazard Ratio.

**Crude:** no adjusted.

**Model 1:** adjusted for age (continuous), gender (male or female).

**Model 2:** adjusted for age(continuous), gender (male or female), race (Mexican American, non-Hispanic White, non-Hispanic Black, Other Hispanic or other races), education (less than high school, high school or above), PIR (continuous), marital (married or non-married), BMI (<30 or ≥30), smoking status (never, former or current), alcohol use (never, former or current).

**Model 3:** adjusted for Model 2 plus CCI (0, 1-3 or >3), antidepressant use (yes or no), depression status (mild, moderate or major).

**Table S9. HR (95% CI)** **for mortality risk with DII (quartiles) among non-cancer and non-CVD depressed participants**

|  | HR (95%CI) | | | | *P* trend |
| --- | --- | --- | --- | --- | --- |
|  | Q1 (<0.27) | Q2 (0.28-1.57) | Q3 (1.58-2.66) | Q4 (≥2.67) |  |
| **All-cause mortality** |  |  |  |  |  |
| Crude | Ref | 0.728 (0.448,1.183) | 1.377 (0.962,1.970) | 1.448 (0.960,2.184) | 0.010 |
| Model1 | Ref | 0.828 (0.519,1.320) | 1.688 (1.174,2.429) | 1.937 (1.336,2.809) | <0.001 |
| Model2 | Ref | 0.822 (0.516,1.310) | 1.379 (0.941,2.020) | 1.498 (1.001,2.243) | 0.012 |
| Model3 | Ref | 0.815 (0.511,1.301) | 1.353 (0.921,1.987) | 1.484 (0.987,2.232) | 0.015 |
| **CVD mortality** |  |  |  |  |  |
| Crude | Ref | 0.968 (0.410,2.282) | 1.504 (0.739,3.062) | 1.326 (0.619,2.842) | 0.254 |
| Model1 | Ref | 1.128 (0.480,2.648) | 1.813 (0.889,3.697) | 1.695 (0.810,3.549) | 0.068 |
| Model2 | Ref | 1.125 (0.471,2.685) | 1.534 (0.671,3.507) | 1.440 (0.630,3.292) | 0.281 |
| Model3 | Ref | 1.162 (0.487,2.774) | 1.577 (0.705,3.526) | 1.431 (0.632,3.238) | 0.278 |
| **Cancer mortality** |  |  |  |  |  |
| Crude | Ref | 1.150 (0.486,2.721) | 0.984 (0.447,2.168) | 1.813 (0.859,3.829) | 0.165 |
| Model1 | Ref | 1.140 (0.492,2.643) | 1.094 (0.494,2.427) | 2.187 (1.082,4.423) | 0.048 |
| Model2 | Ref | 1.355 (0.534,3.438) | 1.255 (0.504,3.123) | 2.543 (1.071,6.039) | 0.055 |
| Model3 | Ref | 1.698 (0.602,4.789) | 1.646 (0.645,4.199) | 2.599 (1.074,6.288) | 0.035 |

**Abbreviations:** DII: dietary inflammatory index; CVD: coronary heart disease; CI: Confidence Intervals; HR: Hazard Ratio.

**Crude:** no adjusted.

**Model 1:** adjusted for age (continuous), gender (male or female).

**Model 2:** adjusted for age(continuous), gender (male or female), race (Mexican American, non-Hispanic White, non-Hispanic Black, Other Hispanic or other races), education (less than high school, high school or above), PIR (continuous), marital (married or non-married), BMI (<30 or ≥30), smoking status (never, former or current), alcohol use (never, former or current).

**Model 3:** adjusted for Model 2 plu s CCI (0, 1-3 or >3), antidepressant use (yes or no), depression status (mild, moderate or major), ln SII (continuous), ln SIRI (continuous).

**Table S10. β (95% CI)** **for systemic inflammatory markers and DII among non-cancer and non-CVD depressed participants**

|  | β | 95% CI | *P* |
| --- | --- | --- | --- |
| **SII** |  |  |  |
| Crude | 0.001 | (0.001, 0.001) | <0.001 |
| Model 1 | 0.001 | (0.001, 0.001) | 0.013 |
| Model 2 | 0.001 | (0.001, 0.001) | 0.009 |
| Model 3 | 0.001 | (0.001, 0.001) | 0.016 |
| **ln SII** |  |  |  |
| Crude | 0.219 | (0.094, 0.343) | 0.001 |
| Model 1 | 0.147 | (0.028, 0.267) | 0.016 |
| Model 2 | 0.153 | (0.044, 0.261) | 0.006 |
| Model 3 | 0.145 | (0.037, 0.254) | 0.009 |
| **SIRI** |  |  |  |
| Crude | 0.026 | (-0.043, 0.095) | 0.461 |
| Model 1 | 0.084 | (0.016, 0.152) | 0.015 |
| Model 2 | 0.063 | (-0.001, 0.126) | 0.054 |
| Model 3 | 0.054 | (-0.009, 0.117) | 0.090 |
| **ln SIRI** |  |  |  |
| Crude | 0.036 | (-0.072, 0.144) | 0.510 |
| Model 1 | 0.126 | (0.021, 0.231) | 0.019 |
| Model 2 | 0.103 | (0.004, 0.201) | 0.041 |
| Model 3 | 0.094 | (-0.004, 0.192) | 0.059 |

**Abbreviations:** SII, systemic immune-inflammation index; SIRI, systemic inflammation response index; ln: natural log-transformed; CI: Confidence Intervals.

**Crude:** no adjusted.

**Model 1:** adjusted for age (continuous), gender (male or female).

**Model 2:** adjusted for age(continuous), gender (male or female), race (Mexican American, non-Hispanic White, non-Hispanic Black, Other Hispanic or other races), education (less than high school, high school or above), PIR (continuous), marital (married or non-married), BMI (<30 or ≥30), smoking status (never, former or current), alcohol use (never, former or current).

**Model 3:** adjusted for Model 2 plu s CCI (0, 1-3 or >3), antidepressant use (yes or no), depression status (mild, moderate or major).

**Table S11. HR (95% CI****) for mortality risk associated with DII levels among non-cancer and non-CVD depressed participants in NHANES 2005-2018**

| Model | HR (95% CI) | *P* | *P* trend |
| --- | --- | --- | --- |
| **All-cause mortality**  **(349/4103)** |  |  |  |
| Crude | 1.117 (1.020,1.223) | 0.017 | 0.010 |
| Model 1 | 1.197 (1.097,1.306) | <0.001 | <0.001 |
| Model 2 | 1.110 (1.012,1.218) | 0.027 | 0.012 |
| Model 3 | 1.108 (1.007,1.218) | 0.035 | 0.015 |
| **CVD mortality**  **(93/4103)** |  |  |  |
| Crude | 1.068 (0.911,1.252) | 0.420 | 0.254 |
| Model 1 | 1.129 (0.959,1.329) | 0.144 | 0.068 |
| Model 2 | 1.071 (0.889,1.291) | 0.468 | 0.281 |
| Model 3 | 1.072 (0.890,1.290) | 0.464 | 0.278 |
| **Cancer mortality**  **(78/4103)** |  |  |  |
| Crude | 1.146 (0.938,1.400) | 0.183 | 0.165 |
| Model 1 | 1.213 (0.995,1.478) | 0.056 | 0.048 |
| Model 2 | 1.256 (0.981,1.608) | 0.071 | 0.055 |
| Model 3 | 1.262 (0.994,1.603) | 0.056 | 0.035 |

**Abbreviations:** DII: dietary inflammatory index; CVD: coronary heart disease; CI: Confidence Intervals; HR: Hazard Ratio.

**Crude:** no adjusted.

**Model 1:** adjusted for age (continuous), gender (male or female).

**Model 2:** adjusted for age(continuous), gender (male or female), race (Mexican American, non-Hispanic White, non-Hispanic Black, Other Hispanic or other races), education (less than high school, high school or above), PIR (continuous), marital (married or non-married), BMI (<30 or ≥30), smoking status (never, former or current), alcohol use (never, former or current).

**Model 3:** adjusted for Model 2 plu s CCI (0, 1-3 or >3), antidepressant use (yes or no), depression status (mild, moderate or major), ln SII (continuous), ln SIRI (continuous).

**Table S12. HR (95****% CI) for mortality risk associated with DII (dichotomous) among non-cancer and non-CVD depressed participants in NHANES 2005-2018**

| Model | HR (95% CI) | | *P* | *P* trend |
| --- | --- | --- | --- | --- |
|  | <1.57 | ≥1.57 |  |  |
| **All-cause mortality**  **(349/4103)** |  |  |  |  |
| Crude | Ref | 1.607 (1.224,2.109) | <0.001 | 0.010 |
| Model 1 | Ref | 1.897 (1.452,2.478) | <0.001 | <0.001 |
| Model 2 | Ref | 1.525 (1.152,2.018) | 0.003 | 0.012 |
| Model 3 | Ref | 1.515 (1.147,2.002) | 0.003 | 0.015 |
| **CVD mortality**  **(93/4103)** |  |  |  |  |
| Crude | Ref | 1.441 (0.843,2.464) | 0.182 | 0.254 |
| Model 1 | Ref | 1.627 (0.933,2.836) | 0.086 | 0.068 |
| Model 2 | Ref | 1.383 (0.742,2.579) | 0.308 | 0.281 |
| Model 3 | Ref | 1.383 (0.751,2.548) | 0.298 | 0.278 |
| **Cancer mortality**  **(78/4103)** |  |  |  |  |
| Crude | Ref | 1.247 (0.748,2.080) | 0.397 | 0.165 |
| Model 1 | Ref | 1.407 (0.841,2.353) | 0.193 | 0.048 |
| Model 2 | Ref | 1.462 (0.803,2.663) | 0.214 | 0.055 |
| Model 3 | Ref | 1.576 (0.861,2.887) | 0.140 | 0.035 |

**Abbreviations:** DII: dietary inflammatory index; CVD: coronary heart disease; CI: Confidence Intervals; HR: Hazard Ratio.

**Crude:** no adjusted.

**Model 1:** adjusted for age (continuous), gender (male or female).

**Model 2:** adjusted for age(continuous), gender (male or female), race (Mexican American, non-Hispanic White, non-Hispanic Black, Other Hispanic or other races), education (less than high school, high school or above), PIR (continuous), marital (married or non-married), BMI (<30 or ≥30), smoking status (never, former or current), alcohol use (never, former or current).

**Model 3:** adjusted for Model 2 plu s CCI (0, 1-3 or >3), antidepressant use (yes or no), depression status (mild, moderate or major), ln SII (continuous), ln SIRI (continuous).

**Table S13. Media****ting effect of systemic inflammatory markers on the association between DII (dichotomous) and all-cause mortality risk among non-cancer and non-CVD depressed participants**

|  | ACME | 95% CI | ADE | PM |
| --- | --- | --- | --- | --- |
| **All-cause mortality** |  |  |  |  |
| **ln SII** |  |  |  |  |
| Crude | 0.00942440 | 0.000522549, 0.02157290 | 0.4016686 | 0.022925 |
| Model 1 | 0.00611541 | -0.001316752, 0.01644541 | 0.4890014 | 0.012351 |
| Model 2 | 0.01085252 | 0.001733007, 0.02363694 | 0.2860116 | 0.036557 |
| Model 3 | 0.01071528 | 0.001369477, 0.02356622 | 0.2729076 | 0.037780 |
| **Male-ln SII** |  |  |  |  |
| Crude | 0.02781674 | 0.00577615, 0.05927986 | 0.4091035 | 0.063665 |
| Model 1 | 0.01529820 | 0.00032457, 0.03883058 | 0.3581947 | 0.040960 |
| Model 2 | 0.01837462 | 0.00107031, 0.04297342 | 0.1254497 | 0.127757 |
| Model 3 | 0.01904387 | 0.00140334, 0.04480626 | 0.1234335 | 0.133662 |
| **Female-ln SII** |  |  |  |  |
| Crude | -0.00042176 | -0.00851850, 0.00637539 | 0.6071548 | -0.000695 |
| Model 1 | -0.00117702 | -0.01517691, 0.01026450 | 0.6529474 | -0.001806 |
| Model 2 | 0.00624823 | -0.00690412, 0.02120928 | 0.4904292 | 0.0125801 |
| Model 3 | 0.00564978 | -0.00698679, 0.02045058 | 0.4574135 | 0.0122009 |
| **ln SIRI** |  |  |  |  |
| Crude | 0.00108905 | -0.02068732, 0.02311062 | 0.4016686 | 0.0027040 |
| Model 1 | 0.01215956 | -0.00213520, 0.02878752 | 0.4890014 | 0.0242628 |
| Model 2 | 0.01276045 | -0.00269087, 0.03128580 | 0.2860116 | 0.0427097 |
| Model 3 | 0.01149796 | -0.00374843, 0.02937778 | 0.2729076 | 0.0404281 |
| **Male-ln SIRI** |  |  |  |  |
| Crude | 0.06136346 | 0.02522072, 0.10795140 | 0.4091035 | 0.1304310 |
| Model 1 | 0.03017337 | 0.00657764, 0.06367181 | 0.3581947 | 0.0776927 |
| Model 2 | 0.02709319 | 0.00402071, 0.06012546 | 0.1254497 | 0.1776103 |
| Model 3 | 0.02702437 | 0.00423194, 0.05901508 | 0.1234335 | 0.1796142 |
| **Female-ln SIRI** |  |  |  |  |
| Crude | -0.01149097 | -0.03765767, 0.01309777 | 0.6071548 | -0.0192910 |
| Model 1 | -0.01065475 | -0.03518331, 0.01187705 | 0.6529474 | -0.0165886 |
| Model 2 | -0.00303141 | -0.02845982, 0.02065988 | 0.4904292 | -0.0062196 |
| Model 3 | -0.00523208 | -0.03049629, 0.01783810 | 0.4574135 | -0.0115707 |

**Abbreviations:** DII: dietary inflammatory index; ACME: average causal mediation effects; ADE: average direct effects; PM: proportion mediated.

**Crude:** no adjusted.

**Model 1:** adjusted for age (continuous), gender (male or female).

**Model 2:** adjusted for age(continuous), gender (male or female; subgroup analysis excluding gender), race (Mexican American, non-Hispanic White, non-Hispanic Black, Other Hispanic or other races), education (less than high school, high school or above), PIR (continuous), marital (married or non-married), BMI (<30 or ≥30), smoking status (never, former or current), alcohol use (never, former or current).

**Model 3:** adjusted for Model 2 plu s CCI (0, 1-3 or >3), antidepressant use (yes or no), depression status (mild, moderate or major), ln SII (continuous), ln SIRI (continuous).

**Table S14. Correlation be****tween DII and blood cell parameters among NHANES 2005-2018 participants with depression**

|  | NEUT | PLA | LYM | MONO |
| --- | --- | --- | --- | --- |
| **All (n = 4981)** |  |  |  |  |
| *R* | 0.077 | 0.095 | 0.073 | 0.032 |
| *P* | <0.001 | <0.001 | <0.001 | 0.023 |
| **Male (n = 2074)** |  |  |  |  |
| *R* | 0.11 | 0.048 | 0.051 | 0.087 |
| *P* | <0.001 | 0.028 | 0.019 | <0.001 |
| **Female (n = 2907)** |  |  |  |  |
| *R* | 0.047 | 0.054 | 0.067 | 0.047 |
| *P* | 0.011 | 0.003 | <0.001 | 0.012 |

**Abbreviations:** DII, dietary inflammatory index; PLA, peripheral platelet count; LYM, lymphocyte count; MONO, monocyte count; NEUT, neutrophil count; NHANES, National Health and Nutrition Examination Survey.

**3. Supplementary figures**


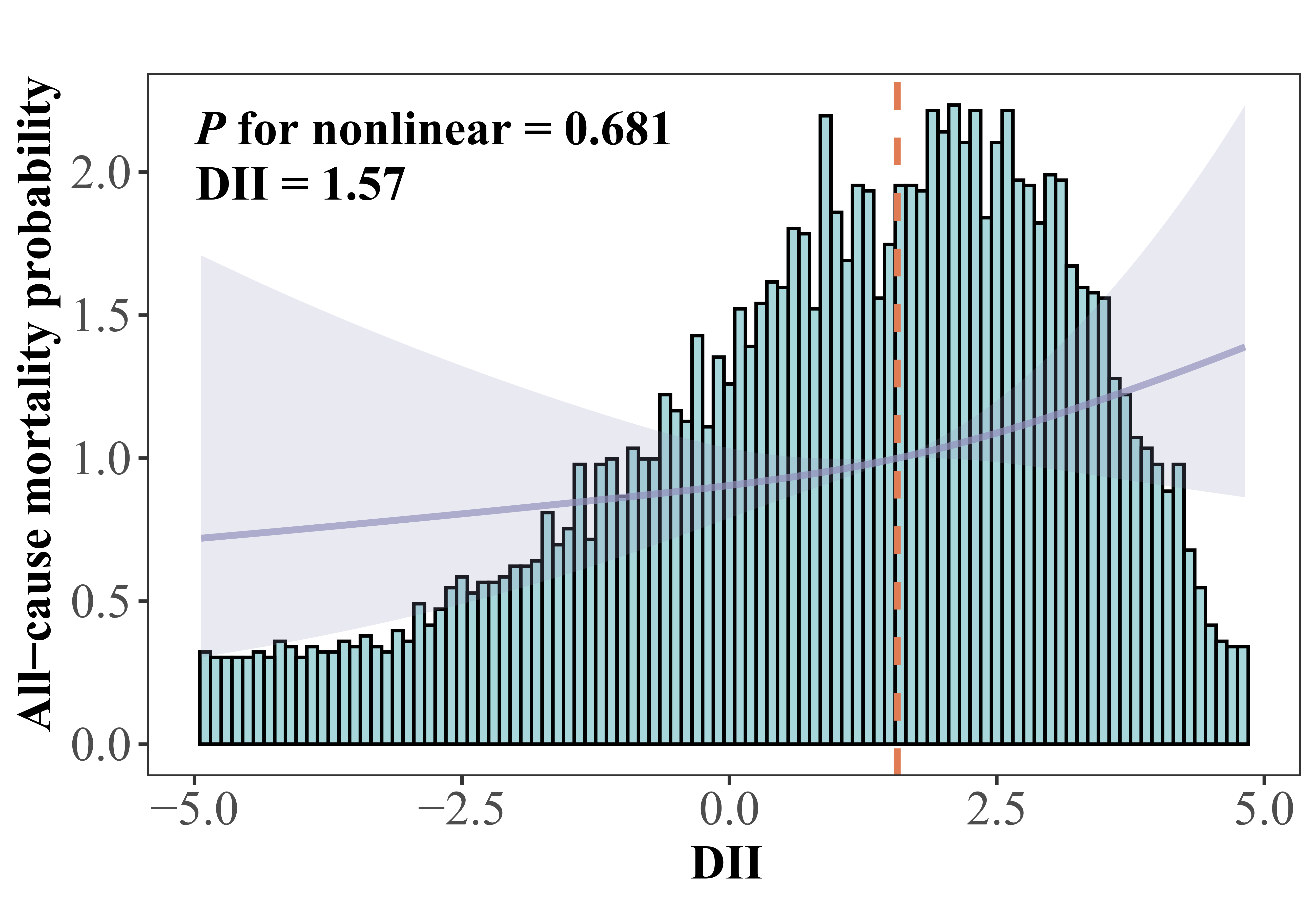
**Figure S1.** Restricted cubic spline model for the association between DII and all-cause mortality risk among non-cancer and non-CVD depressed participants in NHANES 2005-2018. HRs were adjusted for age(continuous), gender (male or female), race (Mexican American, non-Hispanic White, non-Hispanic Black, Other Hispanic or other races), education (less than high school, high school or above), PIR (continuous), marital (married or non-married), BMI (<30 or ≥30), smoking status (never, former or current), alcohol use (never, former or current), CCI (0, 1-3 or >3), antidepressant use (yes or no), depression status (mild, moderate or major), ln SII (continuous); ln SIRI (continuous).


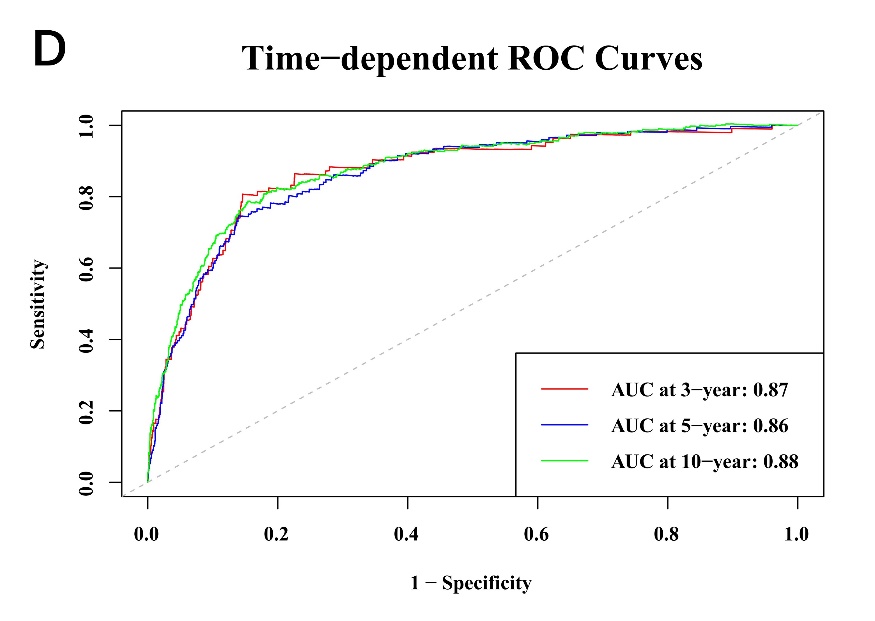

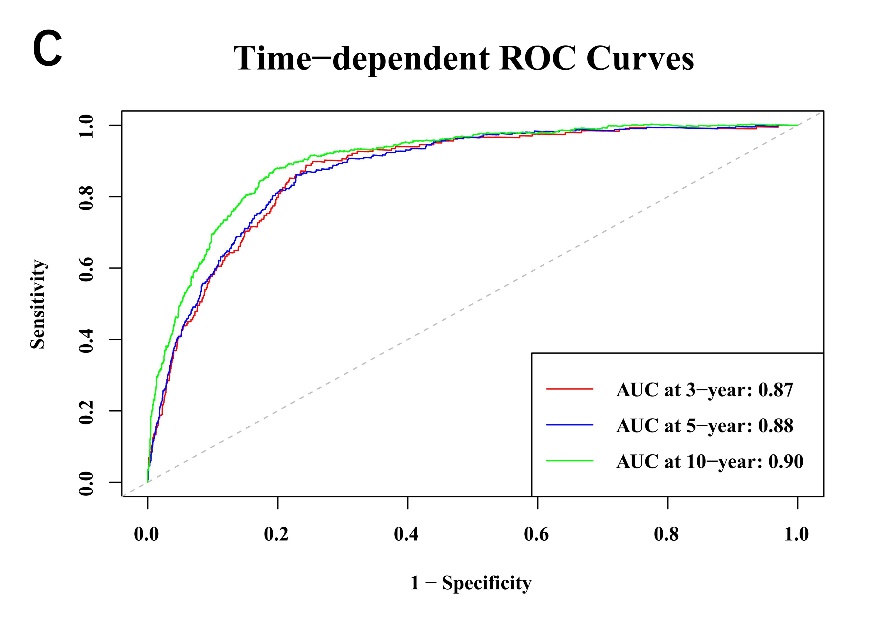

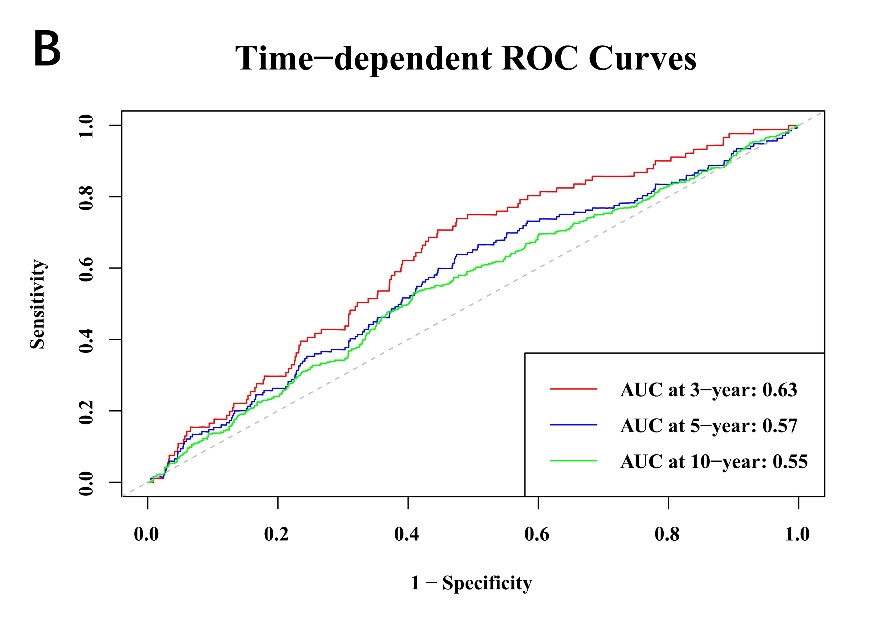

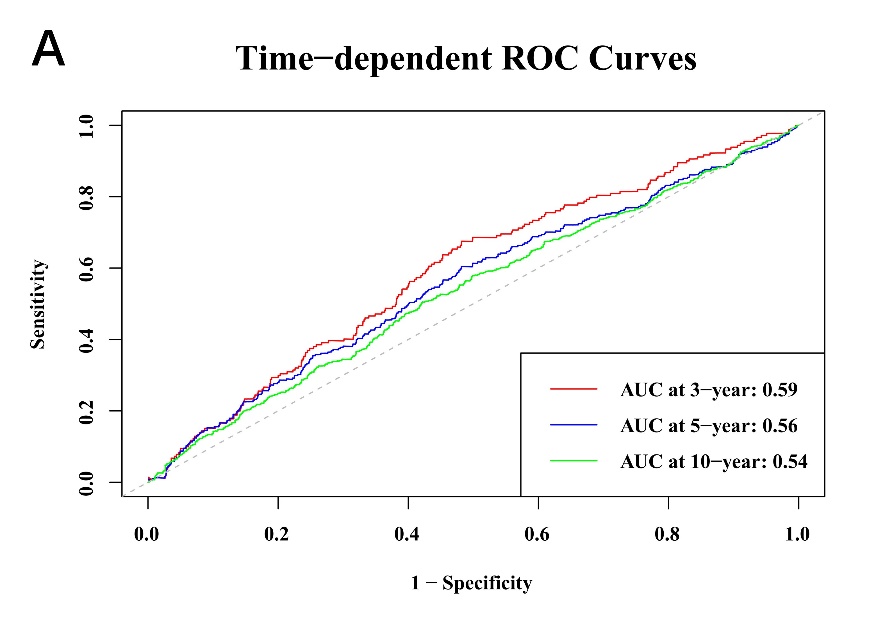
**Figure S2.** ROC curves for time-dependent all-cause mortality risk associated with DII among depression participants in NHANES 2005-2018: (A) ROC curves for 3-year, 5-year, and 10-year all-cause mortality risk; (B) ROC curves for 3-year, 5-year, and 10-year all-cause mortality risk among non-cancer and non-CVD depression participants; (C) ROC curves for 3-year, 5-year, and 10-year all-cause mortality risk with DII adjusted in Model 3; (D) ROC curves for 3-year, 5-year, and 10-year all-cause mortality risk among non-cancer and non-CVD depression participants with DII adjusted in Model 3.


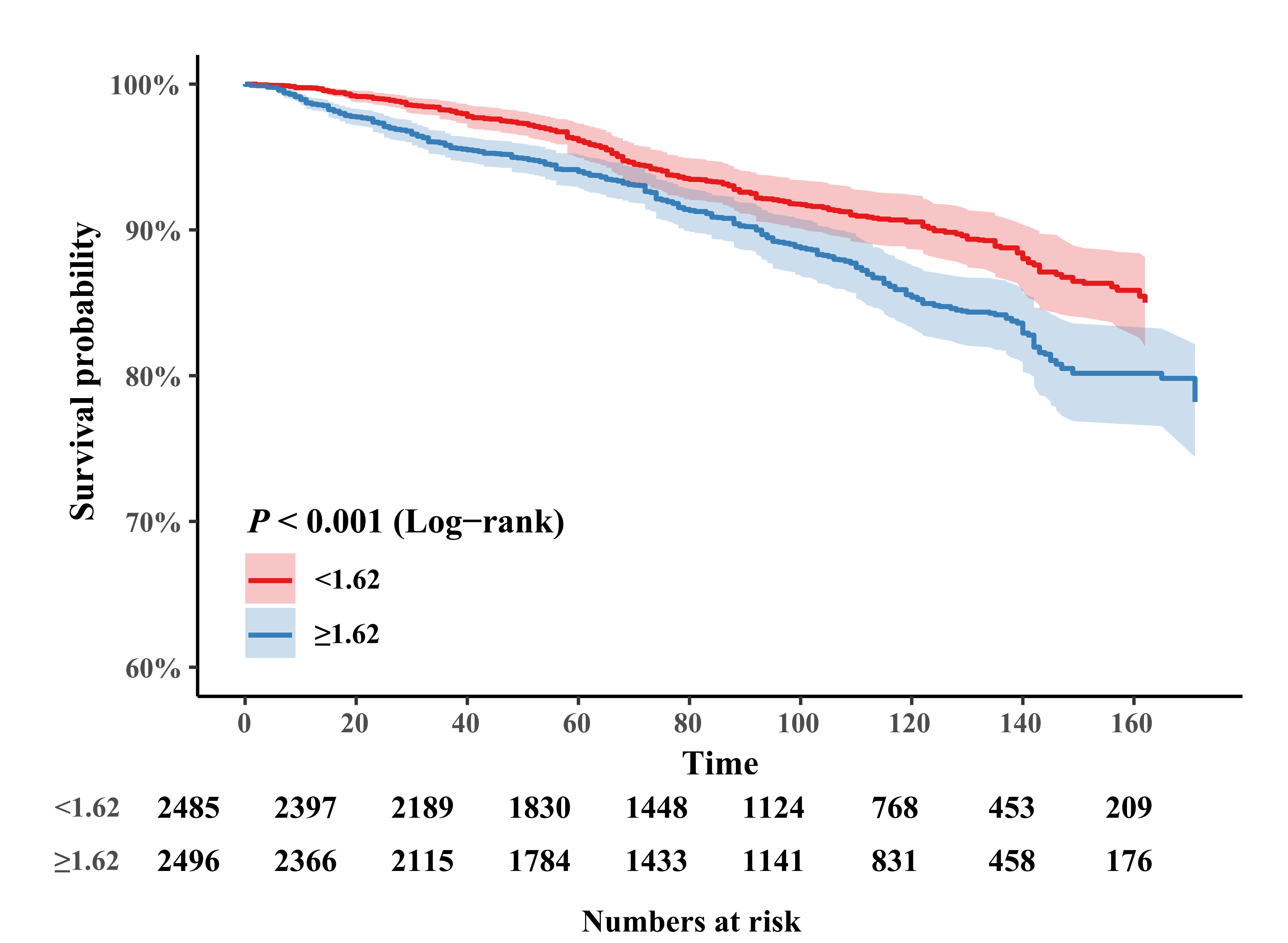
**Figure S3.** Kaplan-Meier curves depicting all-cause mortality risk associated with DII (dichotomous) among non-cancer and non-CVD depressed participants in NHANES 2005-2018.
